# Mas‐Related G Protein‐Coupled Receptor Member D Sustains Hypertension

**DOI:** 10.1002/mco2.70706

**Published:** 2026-03-28

**Authors:** Kun Zhao, Dongxu Hua, Yukang Mao, Xiaoguang Wu, Min Gao, Shidong Song, Lei Chen, Xiangxiang Zheng, Peng Li

**Affiliations:** ^1^ Department of Cardiology The First Affiliated Hospital of Nanjing Medical University Nanjing Jiangsu Province China; ^2^ Duofortunatherapeutic Suzhou Co. Ltd. Suzhou Jiangsu Province China; ^3^ Department of Cardiothoracic Surgery The Second Affiliated Hospital of Soochow University Suzhou Jiangsu Province China; ^4^ Department of Cardiovascular Surgery The First Affiliated Hospital of Nanjing Medical University Nanjing Jiangsu Province China; ^5^ Department of Cardiology Fujian Provincial Hospital, Fuzhou University Affiliated Provincial Hospital Fuzhou, Fujian Province China

**Keywords:** artery remodeling, Ca^2+^/calmodulin‐dependent protein kinase II (CaMKII), hypertension, Mas‐related G protein‐coupled receptor member D (MrgD), voltage‐gated L‐type Ca^2+^ channel (Cav1.2)

## Abstract

Hypertension and its associated complications, including vascular remodeling, pose a major burden on global public health. However, the role of Mas‐related G protein‐coupled receptor member D (MrgD) in hypertension remains incompletely understood. In this study, we observed upregulated MrgD expression in the arterial tissues of hypertensive patients and animal models. In Sprague‐Dawley rats, MrgD overexpression elevated blood pressure (BP) and promoted mesenteric vascular remodeling, whereas MrgD knockdown in spontaneously hypertensive rats normalized BP and ameliorated vascular remodeling. Consistently, MrgD knockout mice exhibited resistance to angiotensin II (Ang II)‐induced hypertension and vascular injury. Mechanistic investigations demonstrated that MrgD facilitated vascular remodeling in vascular smooth muscle cells (VSMCs) through the voltage‐gated L‐type Ca^2^^+^ channel (Cav1.2)‐Ca^2^^+^/calmodulin‐dependent protein kinase IIγ (CaMKIIγ) signaling axis. Co‐immunoprecipitation coupled with mass spectrometry and in vitro functional assays confirmed that Ang II enhanced the interaction among MrgD, CaMKIIγ, and Cav1.2, thereby promoting VSMC phenotypic switch. Through artificial intelligence‐driven screening combined with functional validation, we identified risperidone as a small‐molecule inhibitor of MrgD that effectively attenuated hypertension and vascular remodeling. These findings established MrgD as a key contributor to the pathogenesis of hypertension and underscore its potential as a promising therapeutic target for hypertension and its associated vascular complications.

## Graphical abstract

Targeting MrgD attenuated hypertension and alleviated hypertension‐associated vascular remodeling via repressing Cav1.2‐CaMKIIγ axis. Risperidone, a small‐molecule inhibitor of MrgD, could alleviate hypertension and artery remodeling induced by Ang II infusion.

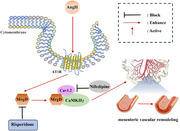

## 1 Introduction

Hypertension represents a pressing global public health challenge and a major contributor to the morbidity and mortality of cardiovascular diseases (CVDs) [1]. Its multifactorial pathogenesis involves a complex interplay of genetic susceptibility, environmental assaults, and physiological dysregulation. Currently, hypertension affects nearly one‐quarter of the global adult population and constitutes a leading risk factor for myocardial infarction, stroke, heart failure, and chronic kidney disease [2]. Beyond the direct hemodynamic burden imposed by elevated blood pressure (BP), vascular remodeling is a central pathological hallmark of hypertension. This process, which exhibits both adaptive and maladaptive features, entails structural and functional changes in the vascular wall, including medial hypertrophy, extracellular matrix deposition, and endothelial dysfunction [3]. Such alterations increase arterial stiffness and reduce vascular compliance, perpetuating a cycle of elevated peripheral resistance that further amplifies BP and damages target organs [3]. Despite significant progress in antihypertensive treatment, particularly through pharmacologic interventions into the renin–angiotensin–system (RAS) and calcium signaling pathways [4, 5], a substantial proportion of patients still exhibit suboptimal BP control, highlighting an urgent need to elucidate deeper molecular mechanisms and identify new targets involved in the pathogenesis of hypertension and its associated vascular complications.

Mechanistically, the RAS plays a pivotal role in maintaining BP homeostasis and regulating vascular physiology, with angiotensin II (Ang II) serving as its principal effector molecule. Ang II exerts potent vasoconstrictive and pro‐fibrotic effects primarily through activating the angiotensin II Type 1 receptor (AT1R), thereby promoting hypertension and vascular remodeling, as demonstrated in both preclinical models and clinical settings [6]. However, the RAS comprises a complex network that includes counter‐regulatory pathways capable of attenuating these pathological effects. Notably, the angiotensin‐(1–7) [Ang‐(1–7)]/Mas receptor (MasR) axis offsets the actions of Ang II by inducing vasodilation, suppressing oxidative stress, and inhibiting fibrotic remodeling [7, 8, 9]. Alamandine (Ala), a heptapeptide, has been recently identified as derived from Ang‐(1–7) via decarboxylation. As an analogous to Ang‐(1–7), Ala exerts antihypertensive and cardioprotective effects [10]. In our previous work, we identified Mas‐related G protein‐coupled receptor member D (MrgD) as a high‐affinity receptor for Ala and demonstrated that Ala mitigates Ang II‐induced cardiac fibrosis through an MrgD‐dependent mechanism [11]. However, despite these insights, the specific role of MrgD in the initiation and progression of hypertension, particularly in vascular remodeling, a key determinant of disease severity, remains largely unexplored.

Vascular smooth muscle cells (VSMCs), core mediators of vascular remodeling, can transit from a contractile to a synthetic and proliferative state, representing a critical event in the pathogenesis of hypertensive vasculopathy [12]. This phenotypic modulation is tightly governed by intracellular calcium signaling, predominantly mediated by voltage‐gated L‐type calcium channels (Cav1.2) [13]. Under physiological conditions, Cav1.2 channels regulate the calcium influx into VSMCs and are essential for maintaining vascular tone [14, 15, 16, 17]. As a hallmark of hypertension, aberrant expression or activity of Cav1.2 increases vascular reactivity and structural remodeling. In line with this, Cav1.2 antagonists, such as nifedipine, are widely used in clinical management of hypertension [18]. Downstream of calcium influx, Ca^2+^/calmodulin‐dependent protein kinase II (CaMKII) serves as a critical integrator of calcium‐dependent signaling in VSMCs [19]. Among the four CaMKII isoforms (α, β, δ, and γ), CaMKIIγ is the predominant isoform expressed in VSMCs and has been reported to negatively regulate VSMC proliferation and vascular remodeling [19]. Nevertheless, CaMKII activation is also implicated in Ang II‐induced hypertension and vascular injury [20, 21]. Although the roles of Cav1.2 and CaMKII in hypertension have been well established, whether MrgD functionally interfaces with this calcium signaling pathway to regulate VSMC behavior and vascular remodeling remains unanswered.

Emerging evidence indicates that G protein‐coupled receptors (GPCRs), including MrgD, function as multifunctional regulators in cardiovascular physiology and represent promising therapeutic targets [22]. As a member of the GPCR superfamily, MrgD is expressed not only in the heart [23] but also in the lungs [24] and brain [25], suggesting its potential involvement in diverse aspects of cardiovascular pathophysiology. However, prior investigations have primarily focused on the interaction between MrgD and Ala in the context of cardiac remodeling [23], whereas its direct role in hypertension and vascular biology remains uncharacterized.

In this study, we utilized a combination of in vivo models and in vitro assays in VSMCs to investigate the effects of MrgD modulation on BP, vascular function, and remodeling processes. Furthermore, we conducted virtual screening using computational approaches to identify small‐molecule inhibitors targeting MrgD and assessed their therapeutic efficacy in mitigating hypertension‐induced vascular injury. By addressing these critical knowledge gaps, our study aimed to establish MrgD as a novel therapeutic target in hypertension and to elucidate its mechanistic role in calcium‐dependent vascular remodeling.

## 2 Results

### 2.1 Effects of MrgD on BP

**FIGURE 1:**
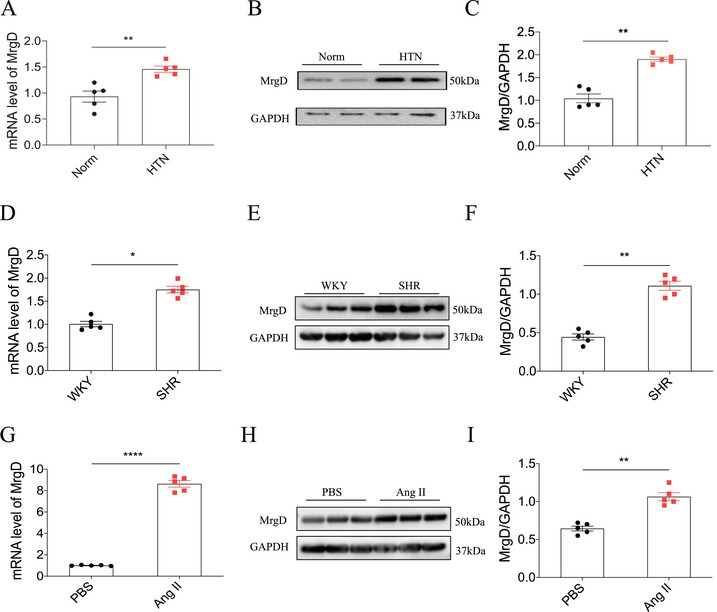
Expression of MrgD under different pathological conditions. (A–C) mRNA (A) and protein (B and C) levels of MrgD in the aorta of HTN patients, *n* = 5 biological replicates per group; (D–F) mRNA (D) and protein (E and F) levels of MrgD in the mesenteric artery of rats, *n* = 5 biological replicates per group; (G–I) mRNA (G) and protein (H and I) levels of MrgD in the VSMCs, *n* = 5 biological replicates per group. The data were expressed as mean ± standard error of the mean (SEM). ^*^ *p* < 0.05, ^**^ *p* < 0.01, ^***^ *p* < 0.001.

**FIGURE 2:**
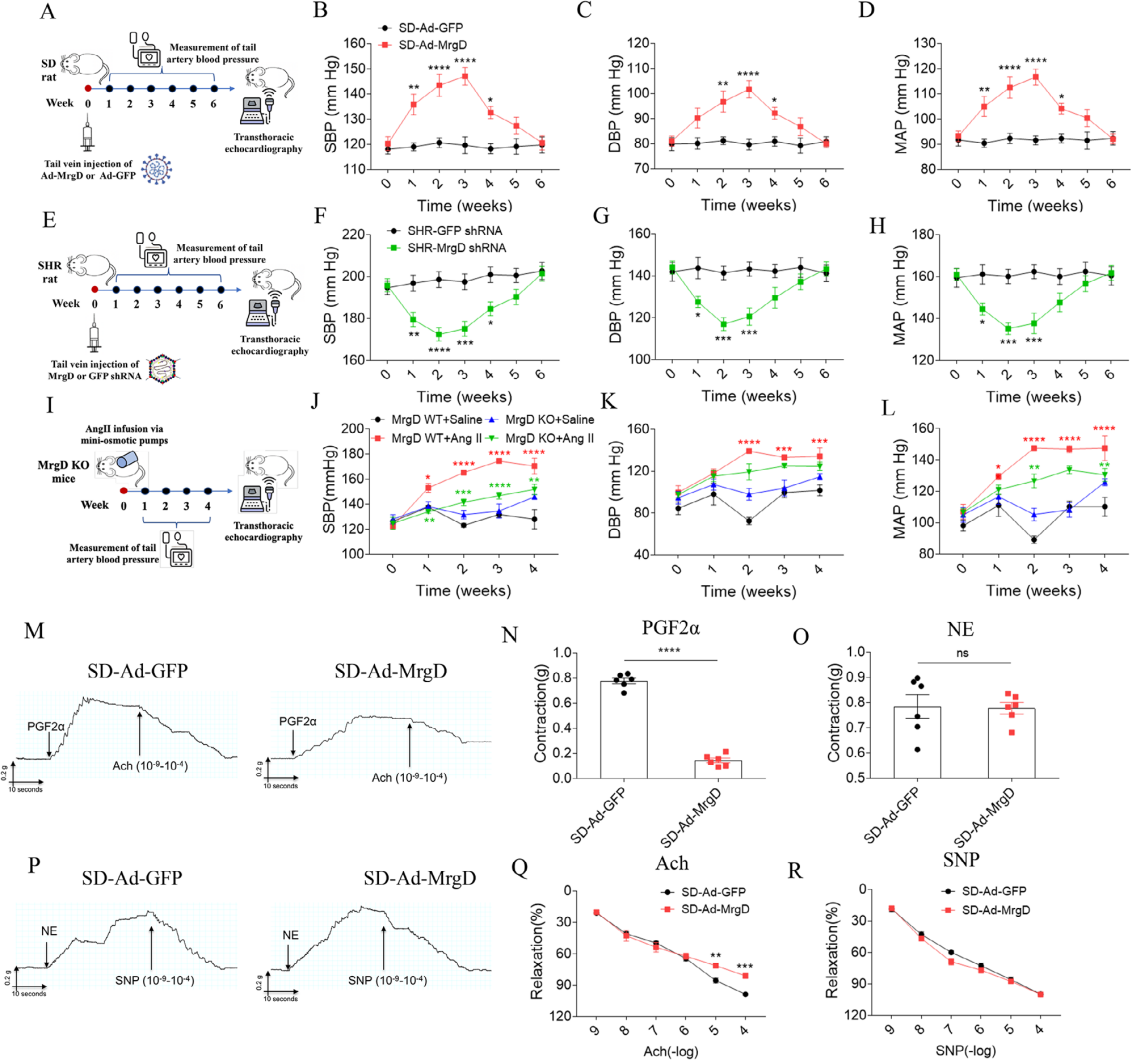
Effect of MrgD on blood pressure. (A) Flow chart of Ad‐MrgD‐treated SD rats; (B–D) the SBP, DBP, and MAP of Ad‐MrgD‐treated SD rats, *n* = 8 biological replicates per group. (E) Flow chart of MrgD shRNA‐treated SHR rats; (F–H) the SBP, DBP, and MAP of MrgD shRNA‐treated SHR rats, *n* = 8 biological replicates per group. (I) Flow chart of Ang II‐treated MrgD KO mice; (J–L) the SBP, DBP, and MAP of Ang II‐treated MrgD KO mice, *n* = 8 biological replicates per group. (M) Representative images of endothelial‐dependent (PFG2α and Ach) vasodilation and vasoconstriction responses. (N) Contraction effect curve of PGF2α‐induced mesenteric arteries, *n* = 6 biological replicates per group; (O) diastolic effect curve of Ach‐induced mesenteric arteries, *n* = 6 biological replicates per group. (P) Representative images of non‐endothelial‐dependent (NE and SNP) vasodilation and vasoconstriction responses. (Q) Contraction effect curve of NE‐induced mesenteric arteries, *n* = 6 biological replicates per group; (R) diastolic effect curve of SNP‐induced mesenteric arteries, *n* = 6 biological replicates per group. The data were expressed as mean ± SEM. ^*^ *p* < 0.05, ^**^ *p* < 0.01, ^***^ *p* < 0.001, ^****^ *p* < 0.0001.

Angiotensin‐converting enzyme (ACE) and AT1R, key components of RAS, are established therapeutic targets for hypertension. Given the known antihypertensive effects of Ala, a RAS‐modulating peptide, we investigated whether its endogenous receptor, MrgD, could serve as a novel target for BP regulation. We first assessed MrgD expression in hypertensive models. MrgD levels were significantly elevated in the aortic tissues of patients with hypertension (Figure 1A–C). Similarly, compared to normotensive Wistar‐Kyoto (WKY) rats, spontaneously hypertensive rats (SHRs) exhibited increased MrgD expression in the mesenteric arteries (Figure 1D–F). In cultured VSMCs, MrgD levels were higher in the Ang II‐treated group in relative to phosphate‐buffered saline (PBS)‐treated controls (Figure 1G–I). To determine the functional impact of MrgD on BP, we employed three distinct animal models. In Sprague‐Dawley (SD) rats, tail vein injection of Adenovirus‐mediated MrgD overexpression (Ad‐MrgD) elevated the systolic BP (SBP), diastolic BP (DBP), and mean arterial pressure (MAP), and these increases were sustained from Week 1 to Week 4 (Figure 2A–D). Correspondingly, MrgD expression was upregulated in the mesenteric artery (Figure S1A) and thoracic aorta (Figure S1B), but not in the renal artery (Figure S1C). Conversely, in SHRs, adenoviral delivery of MrgD‐targeted short hairpin RNA (Ad‐MrgD shRNA) led to a progressive decline in SBP, DBP, and MAP over the same time period (Figure 2E–H). This intervention reduced MrgD expression in the mesenteric artery and thoracic aorta, without affecting levels in the renal artery (Figure S2A, B). Moreover, in MrgD knockout (MrgD KO) mice, the hypertensive response to Ang II infusion was significantly attenuated (Figure 2I–L).

Next, vascular function was evaluated using mesenteric artery rings. Following prostaglandin F2α (PGF2α) stimulation, arterial contractility was significantly reduced in MrgD‐overexpressing SD rats compared to controls (Figure 2M, N). However, we found no significant differences between the two groups in norepinephrine (NE)‐induced vasoconstrictive responses (Figure 2O, P). Furthermore, acetylcholine (Ach)‐induced vasorelaxation was markedly impaired in the MrgD overexpression group (Figure 2Q), whereas the response to sodium nitroprusside (SNP) remained unchanged (Figure 2R).

### 2.2 MrgD Overexpression Induced Mesenteric Artery Remodeling

**FIGURE 3:**
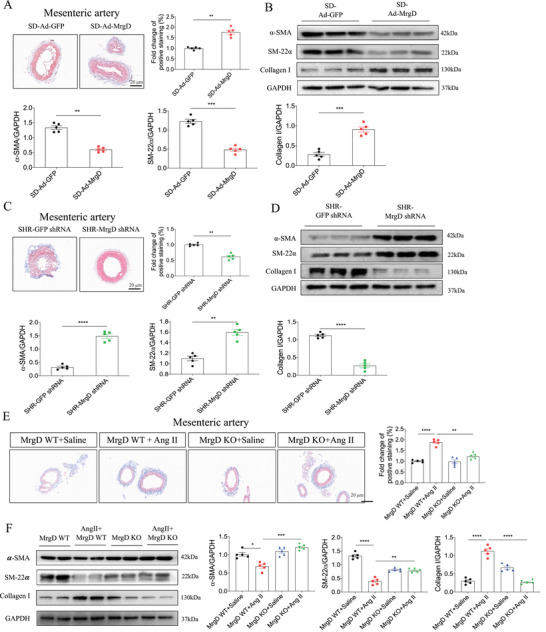
Effects of MrgD expression on remodeling of the mesenteric artery. (A) Masson staining of the mesenteric artery of Ad‐MrgD‐treated SD rats, and the quantitative analysis of fibrosis, *n* = 5 biological replicates per group; (B) protein levels of α‐SMA, SM‐22α, and collagen I in the mesenteric artery of Ad‐MrgD‐treated SD rats, *n* = 5 biological replicates per group; (C) Masson staining of the mesenteric artery of MrgD shRNA‐treated SHR rats, and the quantitative analysis of fibrosis, *n* = 5 biological replicates per group; (D) protein levels of α‐SMA, SM‐22α, and collagen I in the mesenteric artery of MrgD shRNA‐treated SHR rats, *n* = 5 biological replicates per group; (E) Masson staining of the mesenteric artery of Ang II‐treated MrgD KO mice, and the quantitative analysis of fibrosis, *n* = 5 biological replicates per group; (F) protein levels of α‐SMA, SM‐22α, and collagen I in the mesenteric artery of Ang II‐treated MrgD KO mice, *n* = 5 biological replicates per group. The data were expressed as mean ± SEM. ^*^ *p* < 0.05, ^**^ *p* < 0.01, ^***^ *p* < 0.001, ^****^ *p* < 0.0001.

To evaluate the role of MrgD in vascular remodeling, SD rats were administered recombinant Ad‐MrgD via intravenous injection. Pathological staining revealed increased fibrosis in the mesenteric arteries of Ad‐MrgD‐treated rats (Figure 3A), whereas no fibrotic changes were detected in the thoracic aorta or renal arteries (Figure S3A, B). At the molecular level, MrgD overexpression decreased the mRNA expression of the contractile markers α‐smooth muscle actin (α‐SMA) and SM‐22α, and increased the mRNA expression of collagen I in the mesenteric arteries (Figure S3C). These transcriptional changes were not observed in the thoracic aorta following Ad‐MrgD injection (Figure S3D). Protein levels of α‐SMA, SM‐22α, and collagen I in both the mesenteric artery and thoracic aorta mirrored the corresponding mRNA expression profiles (Figure 3B, Figure S3E).

### 2.3 MrgD Downregulation Alleviated Mesenteric Artery Remodeling

To further investigate the protective effects of MrgD silencing against vascular remodeling, SHRs were intravenously injected with adenovirus expressing shRNA‐MrgD (shRNA‐MrgD). Masson's trichrome staining demonstrated that MrgD knockdown significantly reduced the fibrosis in the mesenteric arteries of SHRs, with no evident fibrotic changes in the thoracic aorta or renal arteries (Figure 3C, Figure S4A, B). In the mesenteric arteries, MrgD knockdown increased the mRNA expression of α‐SMA and SM‐22α and decreased collagen I mRNA expression (Figure S4C). No significant transcriptional changes were detected in the thoracic aorta (Figure S4D). Also, protein levels of α‐SMA, SM‐22α, and collagen I in both the mesenteric artery and thoracic aorta followed the same pattern of mRNA changes (Figure 3D, Figure S4E).

### 2.4 MrgD Knockout Alleviated Mesenteric Artery Remodeling

We next established an MrgD KO mouse model and assessed its effects under Ang II stimulation. MrgD deletion significantly attenuated the fibrosis in the mesenteric arteries of Ang II‐treated mice (Figure 3E). The reduction of MrgD expression was presented in the mesenteric arteries of MrgD KO mice (Figure S5A, B). Furthermore, MrgD KO reversed the Ang II‐induced downregulation of contractile markers α‐SMA and SM‐22α, as well as the upregulation of collagen I in the mesenteric arteries (Figure 3F, Figure S5C).

### 2.5 VSMC‐Specific MrgD Overexpression Induced Mesenteric Artery Remodeling

**FIGURE 4:**
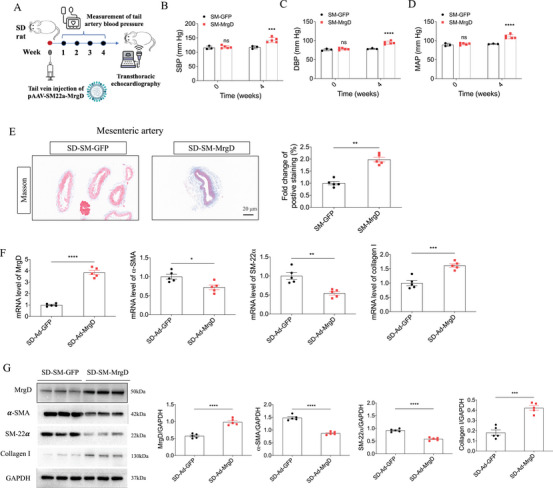
VSMC‐specific overexpressed MrgD induced hypertension and remodeling of the mesenteric artery. (A) Flow chart of VSMC‐specific overexpressed MrgD in SD rats; (B–D) the SBP, DBP, and MAP of rats in two groups, *n* = 6 biological replicates per group. (E) Masson staining of the mesenteric artery, and the quantitative analysis of fibrosis, *n* = 5 biological replicates per group; (F and G) mRNA (F) and protein (G) levels of MrgD, α‐SMA, SM‐22α, and collagen I in the mesenteric artery. The data were expressed as mean ± SEM. ^*^ *p* < 0.05, ^**^ *p* < 0.01, ^***^ *p* < 0.001, ^****^ *p* < 0.0001.

To determine whether MrgD contributes to vascular remodeling through direct effects on VSMCs, we generated a VSMC‐specific MrgD overexpression model (Figure 4A). Selective upregulation of MrgD in VSMCs increased BP in SD rats (Figure 4B–D). Histological analysis revealed that MrgD overexpression in VSMCs significantly promoted the fibrosis in the mesenteric arteries, whereas no fibrotic changes were observed in the renal arteries or thoracic aorta (Figure 4E, Figure S6A–D). Consistently, both mRNA and protein analyses demonstrated that VSMC‐specific MrgD overexpression decreased α‐SMA and SM‐22α and increased collagen I levels in the mesenteric arteries (Figure 4F, G), supporting a role for MrgD in promoting VSMC phenotypic switching and microvascular remodeling in hypertension.

### 2.6 MrgD Played a Key Role in Ang II‐Induced VSMC Remodeling

**FIGURE 5:**
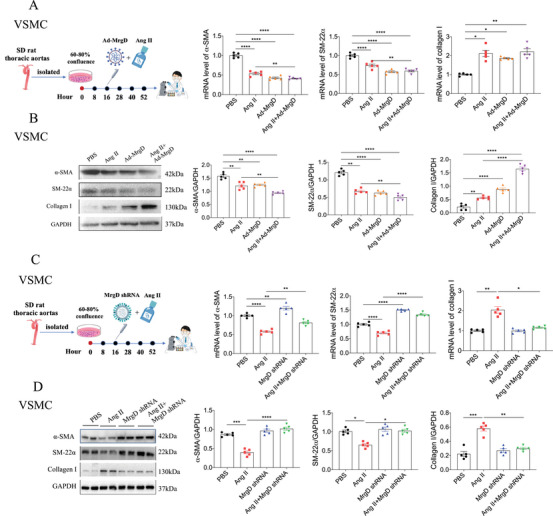
Effects of MrgD expression on VSMCs. (A) Flow chart and mRNA levels of α‐SMA, SM‐22α, and collagen I in Ad‐MrgD‐induced VSMCs treated with/without Ang II from different groups, *n* = 5 biological replicates per group; (B) protein levels of α‐SMA, SM‐22α, and collagen I in Ad‐MrgD‐induced VSMCs treated with/without Ang II from different groups, *n* = 5 biological replicates per group; (C) flow chart and mRNA levels of α‐SMA, SM‐22α, and collagen I in MrgD shRNA‐induced VSMCs treated with/without Ang II from different groups, *n* = 5 biological replicates per group; (D) protein levels of α‐SMA, SM‐22α, and collagen I in MrgD shRNA‐induced VSMCs treated with/without Ang II from different groups, *n* = 5 biological replicates per group. ^*^ *p* < 0.05, ^**^ *p* < 0.01, ^***^ *p* < 0.001, ^****^ *p* < 0.0001.

To further validate the role of MrgD in VSMC remodeling, we conducted a series of in vitro experiments. Initially, we confirmed that MrgD expression was significantly upregulated in VSMCs following adenoviral‐mediated overexpression of MrgD (Figure S7A, B). Either Ang II or Ad‐MrgD decreased α‐SMA and SM‐22α, and increased collagen I mRNA levels (Figure 5A). Consistently, α‐SMA and SM‐22α protein levels were reduced, whereas the collagen I protein level was elevated in VSMCs subjected to Ang II or Ad‐MrgD treatment. Notably, Ad‐MrgD further enhanced these Ang II‐induced changes by further decreasing α‐SMA and SM‐22α and increasing collagen I expression (Figure 5B). Conversely, MrgD expression was effectively suppressed in VSMCs following adenoviral‐mediated knockdown (Ad‐MrgD shRNA) (Figure S7C, D). In Ang II‐treated VSMCs, the downregulation of α‐SMA and SM‐22α and upregulation of collagen I at the mRNA level were reversed by MrgD knockdown (Figure 5C). Similarly, the changes in corresponding protein expression induced by Ang II were also reversed following Ad‐MrgD shRNA treatment (Figure 5D).

### 2.7 MrgD Induced Arterial Remodeling via the Cav1.2‐CaMKIIγ Pathway

**FIGURE 6:**
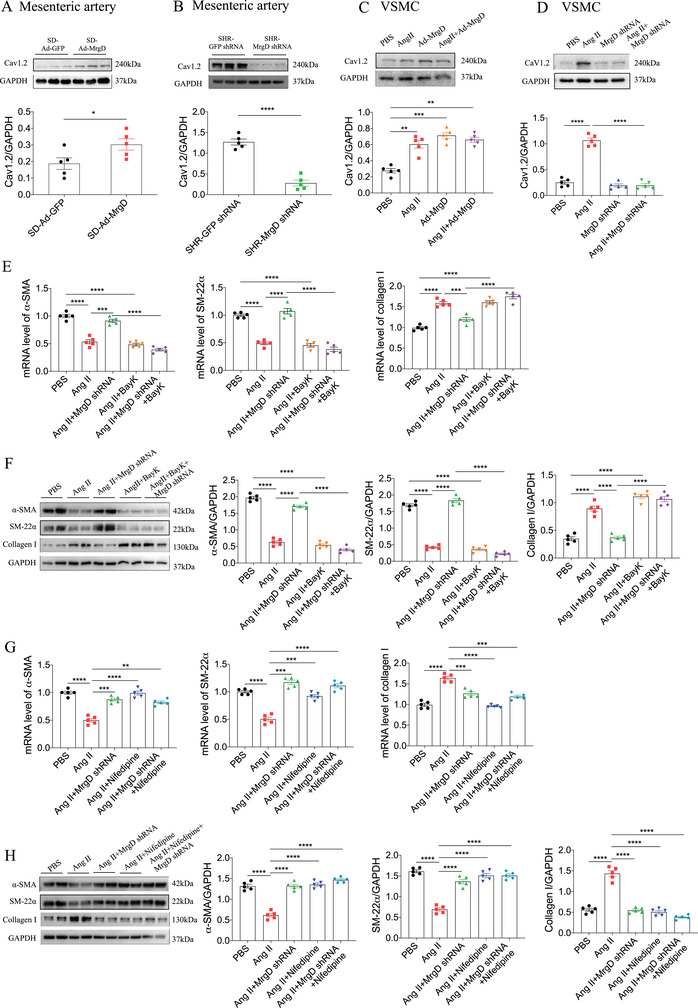
MrgD induced artery remodeling via Cav1.2. (A) MrgD overexpression increased Cav1.2 protein levels in the mesenteric artery of SD rats, *n* = 5 biological replicates per group; (B) MrgD knockdown reduced Cav1.2 protein levels in the mesenteric artery of SHR rats, *n* = 5 biological replicates per group; (C) Ang II or Ad‐MrgD treatment increased Cav1.2 protein levels in the VSMCs, *n* = 5 biological replicates per group; (D) MrgD shRNA treatment reduced Ang II‐induced Cav1.2 protein levels in the VSMCs, *n* = 5 biological replicates per group; (E and F) BayK inhibited the reversing effects of MrgD knockdown on the decreases of α‐SMA and SM‐22α, and the increase of collagen I mRNA (E) and proteins (F) in the VSMCs induced by Ang II, *n* = 5 biological replicates per group; (G and H) nifedipine reversed the decreases of α‐SMA and SM‐22α, and the increase of collagen I mRNA (G) and proteins (H) in the VSMCs induced by Ang II, *n* = 5 biological replicates per group. ^*^ *p* < 0.05, ^**^ *p* < 0.01, ^***^ *p* < 0.001, ^****^ *p* < 0.0001.

Cav1.2 channels, located on the plasma membrane of VSMCs, play a pivotal role in the regulation of BP, vascular tone, and arterial remodeling [26]. To determine whether MrgD mediates arterial remodeling through Cav1.2, we assessed Cav1.2 expression following MrgD modulation in vivo and in vitro. In SD rats, adenoviral‐mediated overexpression of MrgD led to a significant increase in Cav1.2 mRNA (Figure S8A) and protein (Figure 6A) expression in the mesenteric arteries, whereas no changes were detected in the thoracic aorta (Figure S8B) or renal arteries (Figure S8C). Conversely, in SHRs, MrgD knockdown reduced Cav1.2 mRNA (Figure S8D) and protein (Figure 6B) expression in the mesenteric arteries, without affecting their levels in the thoracic aorta (Figure S8E) or renal arteries (Figure S8F). In cultured VSMCs, both Ang II and Ad‐MrgD treatment increased Cav1.2 expression at the mRNA (Figure S9A) and protein (Figure 6C) levels. This upregulation was effectively reversed by Ad‐MrgD shRNA‐mediated MrgD knockdown at both the transcriptional (Figure S9B) and translational (Figure 6D) levels.

Functionally, the L‐type calcium channel agonist BayK abrogated the reversal effects of MrgD knockdown, restoring the Ang II‐induced downregulation of α‐SMA and SM‐22α, as well as the upregulation of collagen I mRNA (Figure 6E) and protein (Figure 6F). In contrast, the L‐type calcium channel blocker nifedipine counteracted the effects of Ang II, thereby reversing the reduction in α‐SMA and SM‐22α expression and suppressing the upregulation in collagen I expression (Figure 6G, H) in VSMCs.

**FIGURE 7:**
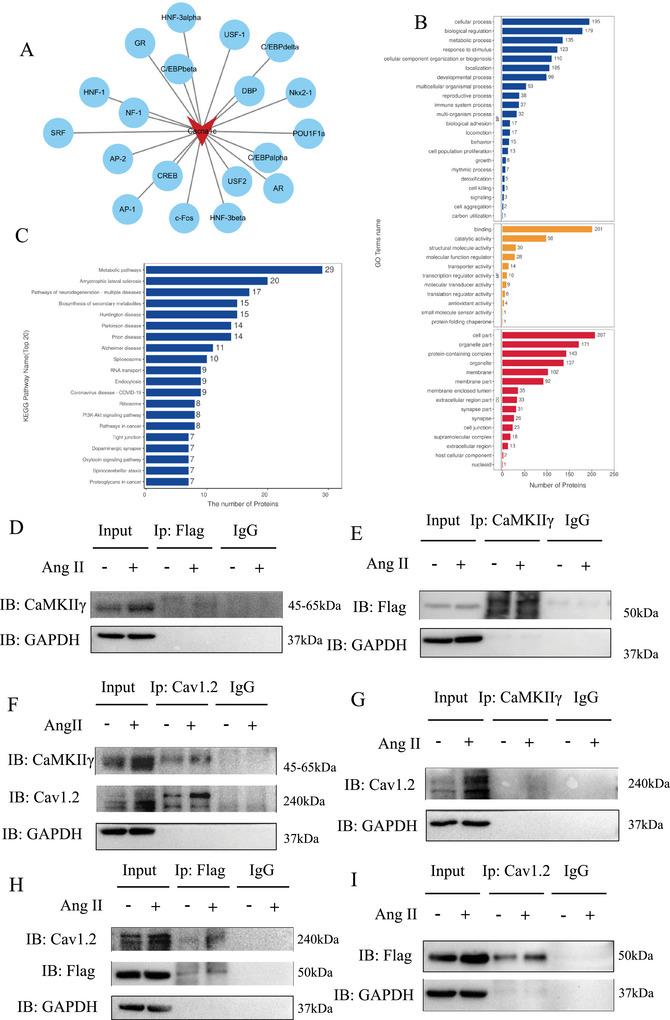
CoIP‐MS mass spectrometry and JASPAR predicted transcription factors of Cav1.2. (A) 19 transcription factors of Cav1.2 predicted by JASPAR, including GR, c‐fos, C/EBPbeta, C/EBPalpha, HNF‐3alpha, USF2, DBP, NF‐1, CREB, C/EBPdelta, HNF‐3beta, Nkx2‐1, SRF, AP‐1, POU1F1a, USF‐1, HNF‐1, AR, and AP‐2; (B and C) gene ontology (GO) terms and Kyoto Encyclopedia of Genes and Genomes (KEGG) pathways enriched in the 19 transcription factors of Cav1.2. The JASPAR software (http://jaspar.genereg.net/) was used to predict the transcription factors of Cav1.2 (relative score ≥ 0.85) following the previous instruction; (D and E) Co‐IP experiments showed that MrgD interacted directly with CaMKIIγ; (F and G) Ang II‐induced CaMKIIγ and Cav1.2 expression in the VSMCs; (H and I) Ang II induced MrgD to co‐immunoprecipitate with Cav1.2.

Using the JASPAR database, a total of 19 candidate transcription factors were predicted to regulate Cav1.2 expression, including glucocorticoid receptor (GR), c‐Fos, CCAAT/enhancer‐binding protein β (C/EBPβ), C/EBPα, hepatocyte nuclear factor‐3α (HNF‐3α), upstream stimulatory factor 2 (USF2), DBP, nuclear factor 1 (NF‐1), cAMP response element‐binding protein (CREB), C/EBPδ, HNF‐3β, Nkx2‐1, serum response factor (SRF), activator protein 1 (AP‐1), POU Class 1 homeobox 1a (POU1F1a), USF1, HNF‐1, androgen receptor (AR), and AP‐2 (Figure 7A–C). However, co‐immunoprecipitation coupled with mass spectrometry (CoIP‐MS) revealed that none of these transcription factors interacted with MrgD, indicating that MrgD does not participate in the transcriptional regulation of Cav1.2.

Interestingly, CoIP‐MS analysis identified the presence of CaMKIIγ in Ang II‐treated VSMCs, but not in PBS‐treated controls, suggesting the potential formation of an MrgD‐CaMKIIγ‐Cav1.2 complex. To validate this interaction, VSMCs were transfected with Flag‐tagged Ad‐MrgD and subsequently treated with either Ang II or PBS. Co‐immunoprecipitation (Co‐IP) was performed using Flag‐labeled MrgD protein to determine the complex formation with CaMKIIγ and Cav1.2. The results demonstrated that MrgD directly interacted with CaMKII (Figure 7D, E). Furthermore, Ang II stimulation upregulated CaMKIIγ and Cav1.2 expression in VSMCs (Figure 7D, F) and enhanced the Co‐IP of both CaMKII and MrgD (Figure 7F–I) with Cav1.2. These findings suggest that Ang II promotes the assembly of an MrgD‐CaMKIIγ‐Cav1.2 signaling complex, thereby facilitating the upregulation of Cav1.2 expression.

### 2.8 MrgD Small‐Molecule Inhibitor Alleviated Mesenteric Artery Remodeling

**FIGURE 8:**
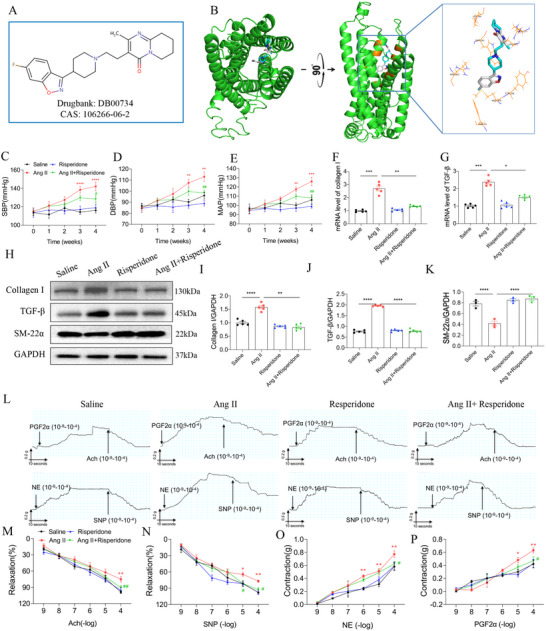
Risperidone alleviated hypertension and remodeling of the mesenteric artery. (A) The structure of risperidone; (B) the structure of risperidone binding with MrgD; (C–E) risperidone relieved Ang II‐induced hypertension in mice, ^*^vs. Saline and ^#^vs. Ang II, *n* = 8 biological replicates per group. (F and G) Risperidone reversed Ang II‐induced increases in the mRNA levels of collagen I and TGF‐β in the mesenteric artery of mice, *n* = 5 biological replicates per group. (H–K) Risperidone reversed Ang II‐induced changes in the protein levels of collagen I, TGF‐β, and SM‐22α in the mesenteric artery of mice, *n* = 5 biological replicates per group. (L) Representative images of endothelial‐dependent (PFG2α and Ach) and non‐endothelial‐dependent (NE and SNP) vasodilation and vasoconstriction responses; (M and N) risperidone reduced vasorelaxation in the mesenteric artery of mice treated with Ang II, ^*^vs. Saline and ^#^ vs. Ang II, *n* = 6 biological replicates per group. (O and P) Risperidone reduced contraction in the mesenteric artery of mice treated with Ang II, ^*^vs. Saline and ^#^vs. Ang II, *n* = 6 biological replicates per group. The data were expressed as mean ± SEM. ^*/#^ *p* < 0.05, ^**/##^ *p* < 0.01, ^***^ *p* < 0.001, ^****^ *p* < 0.0001.

To identify potential small‐molecule inhibitors targeting MrgD, we conducted virtual screening based on a library of U.S. Food and Drug Administration (FDA)‐approved compounds. A total of 22 candidate inhibitors were identified (Table S1). Among these, risperidone was validated for its efficacy in reversing Ang II‐induced phenotypic switching in VSMCs (Figure S10). The molecular structure of risperidone is shown in Figure 8A. Molecular docking analysis revealed that risperidone could bind to MrgD through eight key amino acid residues: F75, M105, Y106, R177, V181, W241, L244, and R262 (Figure 8B). In vivo, risperidone treatment significantly attenuated Ang II‐induced hypertension in mice (Figure 8C–E). Moreover, risperidone effectively reversed Ang II‐induced mesenteric artery remodeling. Specifically, risperidone suppressed Ang II‐induced changes of collagen I, TGF‐β, and SM‐22α levels (Figure 8F–K) in mesenteric arteries. Functionally, risperidone enhanced Ach‐induced and SNP‐induced vasorelaxation in mesenteric arteries compared to the Ang II group (Figure 8L–N). Concurrently, it mitigated the vasoconstrictive responses to NE (Figure 8O) and PGF2α (Figure 8P).

## 3 Discussion

Although previous studies have demonstrated that Ala exerts antihypertensive effects, the precise role of its endogenous receptor, MrgD, in the initiation and progression of hypertension has remained unclear. In the present study, we provided experimental evidence that MrgD knockdown confers protective effects against hypertension and mesenteric vascular remodeling. These effects appear to be mediated via the Cav1.2‐CaMKIIγ signaling pathway, thereby confronting MrgD as a potential therapeutic target for future clinical intervention.

Hypertension affects approximately one‐quarter of the global adult population and is responsible for up to 400,000 deaths annually [27]. Despite the high burden of this disease, no new antihypertensive agents have been approved by the FDA over the past decade [28]. Current pharmacological treatments are often challenged by limited efficacy and off‐target organ toxicity, highlighting the urgent need for novel therapeutic strategies. The RAS is a core regulator of BP and fluid balance [29]. Key bioactive components of RAS, including Ang II and aldosterone, exert classical physiological effects, such as vasoconstriction, sodium retention, and tissue remodeling, while also promoting inflammation and fibrosis [30]. Beyond the traditional ACE/Ang II/AT1R axis, a counter‐regulatory pathway composed of angiotensin‐converting enzyme 2 (ACE2), Ang‐(1–7), and the MasR plays a protective role in cardiovascular regulation [31, 32]. Ang‐(1–7), as the principal ligand for MasR, has demonstrated a significant therapeutic efficacy in the experimental models of hypertension [33]. As a novel heptapeptide within the non‐classical RAS, Ala is generated from Ang‐(1–7) through decarboxylation and exerts vasodilatory effects in SHRs via binding to MrgD. MrgD, a shared receptor by both Ang‐(1–7) and Ala, is expressed in arterial SMCs, cardiac tissue, and atherosclerotic plaques [34], suggesting that it may participate in multiple pathophysiological processes associated with CVDs. To date, however, only our previous study has demonstrated that Ala, acting as an inhibitory ligand of MrgD, attenuates Ang II‐induced cardiac remodeling [23]. No studies have yet comprehensively examined the specific role of MrgD in the context of hypertension or other CVDs. Nonetheless, given the physiological relevance of Ala and its receptor MrgD within the cardiovascular system, both may serve as promising targets for the development of next‐generation antihypertensive and cardioprotective therapies.

In this study, we demonstrated that silencing MrgD effectively alleviates Ang II‐induced mesenteric artery remodeling. Vascular injury and repair typically involve fibrotic changes in both the medial and adventitial layers. Previous research has established a strong association between vascular remodeling and the alteration of smooth muscle contractility in the thoracic aorta of hypertensive mice [35]. Consistent with these findings, our data revealed that arteries from MrgD‐overexpressing SD rats exhibited a diminishment of contractile force.

To further validate the role of MrgD in vascular remodeling, we utilized a rat model with VSMC‐specific overexpression of MrgD. In addition, we examined MrgD expression in human umbilical vein endothelial cells (HUVECs) and human coronary artery endothelial cells (HCAECs). Both cell types showed detectable levels of MrgD expression, which was further confirmed in mesenteric arteries by immunofluorescent co‐staining for CD31 and MrgD (Figure S11). These findings suggest that endothelial MrgD expression may contribute to the differential vasodilatory responses following Ach and SNP stimulation.

Notably, while Ala exhibits antihypertensive properties, overexpression of MrgD imparts a pressor effect. Our previous work supports this distinction, demonstrating that Ala attenuates myocardial hypertrophy and fibrosis in cells overexpressing MrgD [23]. These findings support the notion that Ala acts as an antagonist, rather than an agonist, on the MrgD receptor. The same relationship was also observed between the traditional antihypertensive drug losartan and its receptor AT1R [36]. As a competitive AT1R blocker, losartan was confirmed to increase AT1R abundance in the rat left ventricle via a negative feedback mechanism [36].

Notably, although BP levels returned to baseline by the 6‐week endpoint in both MrgD‐overexpressing and MrgD KO rats, our histopathological and molecular analyses strongly suggest that the mesenteric artery remodeling was driven by acute, transient fluctuations in BP induced by altered MrgD expression. To further explore the role of MrgD in sustained hypertension‐related vascular injury, we conducted complementary endpoint experiments in MrgD KO mice with or without Ang II infusion. The results clearly demonstrated that MrgD expression could be modulated to attenuate mesenteric artery remodeling under conditions of chronic hypertension. Collectively, these findings underscore the dual role of MrgD in vascular remodeling, implicating it in both acute BP fluctuations and chronic hypertensive states. It is important to emphasize that persistent elevation of BP can surpass the adaptive capacity of the vascular wall, thereby promoting maladaptive remodeling. This pathological process contributes to luminal narrowing and high peripheral vascular resistance, which in turn further elevates BP, perpetuating a vicious cycle that exacerbates vascular damage [37].

Multiple studies have demonstrated that Cav1.2 channel activity is enhanced in the mesenteric, femoral, and cerebral arteries of SHRs and Ang II‐infused mice. Persistent upregulation of Cav1.2 expression has been closely associated with channel hyperactivity in VSMCs [38]. Consistently, our findings showed that overexpression of MrgD led to upregulation of Cav1.2 expression both in vivo and in vitro. Alterations in ion channel function, particularly Ca^2^^+^ channels in VSMCs, exert profound effects on the development and progression of hypertension [39]. Under pathological conditions, activation of vascular Cav1.2 channels contributes to vascular dysfunction and cardiac hypertrophy in several animal models [40, 41]. Intracellular free Ca^2^^+^ serves not only as a key second messenger in the regulation of BP [42] but also as a molecular effector driving the phenotypic transition of VSMCs. Notably, Ca^2^^+^ influx mediated by Cav1.2 channels has been shown to induce vascular contraction and activate the transcription of downstream target genes, thereby supporting both the contractile function and phenotypic maintenance of VSMCs. Interestingly, Ca^2^^+^ influx is also regarded as a compensatory mechanism that sustains elevated vascular tone during hypertension [39]. Encouragingly, our data indicate that MrgD promotes arterial remodeling and contributes to the development of hypertension through a mechanism involving Cav1.2 channel activation.

Moreover, although no previous studies have directly linked MrgD silencing to Cav1.2 channel activity, multiple GPCRs and their corresponding ligands have been shown to regulate Cav1.2 in VSMCs and cardiomyocytes [43, 44, 45]. Notably, β‐alanine, an established ligand of MrgD, has been reported to modulate vascular tone by promoting calcium influx [46]. While risperidone is known to influence cytosolic Ca^2^^+^ release and uptake [47, 48], it does not affect voltage‐dependent K^+^ or L‐type Ca^2^^+^ currents in GH(3) cells at concentrations up to 10 µM [49]. Previous research has identified the Cav1.2‐mediated CaMKII signaling pathway as a proximal regulatory mechanism in CVDs [50, 51]. CaMKII contains two Ca^2^^+^‐binding domains that are essential for transducing intracellular calcium signals in vascular biology. Functionally, CaMKII acts as a key integrator that converts dynamic changes in intracellular Ca^2^^+^ concentrations into a spectrum of biological responses, thereby allowing the initiation or progression of cardiovascular and other diseases [52, 53]. Among the four known CaMKII isoforms (α, β, δ, and γ), CaMKIIβ and CaMKIIγ are predominantly expressed in VSMCs and play distinct roles in calcium homeostasis [54, 55]. CaMKIIβ has been found to promote VSMC proliferation and migration in vitro [56, 57] and exacerbate vascular remodeling following injury in vivo [58]. In contrast, CaMKIIγ functions as a negative regulator of VSMC proliferation and can counteract CaMKIIβ‐mediated vascular remodeling [59].

Previous studies have demonstrated that CaMKII takes on multifaceted profiles in signal integration during Ang II‐induced hypertension [60]. CaMKII becomes activated following vascular injury, including injury associated with hypertensive states [61]. Notably, both pharmacological and genetic inhibition of CaMKII effectively suppress Ang II‐induced hypertension and prevent vascular remodeling in large vessels such as the aorta, as well as in resistance arteries, including the mesenteric artery [55, 61, 62]. In the present study, we found that Ang II upregulates Cav1.2 expression by enhancing the interaction between MrgD and CaMKIIγ, thereby facilitating their binding to Cav1.2.

To explore the clinical relevance of these findings, we virtually screened an FDA‐approved small‐molecule drug library and identified risperidone as a potential MrgD inhibitor. Risperidone is a widely prescribed second‐generation antipsychotic agent with a well‐established safety and efficacy profile in the treatment of psychotic disorders [63, 64]. Our functional experiments confirmed that risperidone attenuated Ang II infusion‐induced hypertension and arterial remodeling. These findings suggest that pharmacological targeting of MrgD with risperidone may represent a promising therapeutic strategy for hypertension and associated vascular remodeling.

### 3.1 Limitation

This study was conducted exclusively in male animal models. This design was intended to minimize the confounding effects of sex hormones and to establish a preliminary proof‐of‐concept for the role of MrgD in hypertension. However, the use of a single‐sex cohort limits the assessment of sex‐specific effects. Our primary goal was to establish a clear mechanistic link between MrgD and hypertension‐related vascular remodeling, without the added complexity of hormone‐dependent variability. For this reason, many foundational studies in this field have similarly employed male animal models [65, 66, 67, 68]. Nonetheless, given the well‐documented sex‐related differences in clinical hypertension [69], future studies should incorporate both male and female models to evaluate the generalizability of our findings and to determine the extent of sexual dimorphism. Second, the clinical correlation was based on a limited number of human artery samples. Finally, while risperidone showed efficacy, its pleiotropic effects necessitated future studies with more specific MrgD antagonists to establish its therapeutic relevance.

## 4 Conclusion

Our findings demonstrate that silencing MrgD, potentially achievable through pharmacological inhibition with risperidone, could attenuate hypertension and mitigate hypertension‐associated vascular remodeling by modulating the Cav1.2‐CaMKIIγ signaling axis. These findings provide mechanistic insights and lay a foundation for the development of novel therapeutic strategies targeting MrgD against hypertension.

## 5 Materials and Methods

### 5.1 Human Arterial Tissue Samples

Arterial specimens were obtained from adult normotensive (Norm; *n* = 5) and hypertensive (HTN; *n* = 5) patients undergoing surgery at The First Affiliated Hospital of Nanjing Medical University (Nanjing, China). The study protocol was approved by the Institutional Review Board of The First Affiliated Hospital of Nanjing Medical University (Approval No. 2019‐SR‐067). Demographic and clinical characteristics of the enrolled participants are summarized in Table S2.

### 5.2 Animal Ethics

All animal experiments were conducted in accordance with the Guide for the Care and Use of Laboratory Animals (NIH Publication No. 85‐23, revised 1996) and approved by the Institutional Animal Care and Use Committee of Nanjing Medical University. Animals were maintained in a temperature‐controlled facility on a 12‐h light/12‐h dark cycle, with ad libitum access to standard chow and tap water.

### 5.3 Tail Arterial BP Measurement

Tail arterial BP, including SBP, DBP, and MAP, was measured weekly in conscious male rats, specifically SD, SHRs, and WKY rats (160–180 g; purchased from Beijing Vital River Laboratory Animal Technology Co. Ltd.), using a noninvasive computerized tail‐cuff system (Kent Scientific Corporation, CT, USA) (*n* = 8 per group). Prior to each measurement, animals were placed in a temperature‐controlled chamber at 28°C for 10–20 min to stabilize tail blood flow for accurate pulse detection. To reduce measurement variability due to stress, rats underwent daily acclimatization training for at least 7 consecutive days before the start of the experimental protocol. Final BP readings were derived by averaging 8–12 independent measurements per rat [70].

### 5.4 MrgD Overexpression

Male SD rats were used to assess the effects of MrgD overexpression on BP regulation. Ad‐MrgD and a control adenovirus expressing green fluorescent protein (Ad‐GFP) were obtained from Genechem (Shanghai, China). Each rat received a tail vein injection of Ad‐MrgD or Ad‐GFP at a titer of 1 × 10^7^ transduction units (TU)/mL. Tail arterial BP was monitored weekly over a 6‐week period (*n* = 8 per group). At the study endpoint, the rats were euthanized via cervical dislocation under 3.5% isoflurane anesthesia for tissue collection. Transfection efficiency was confirmed by assessing MrgD expression levels in vascular tissues.

To achieve vascular smooth muscle‐specific overexpression of MrgD, a recombinant adeno‐associated virus (AAV) vector (pAAV‐SM22αp‐MrgD‐3×FLAG‐P2A‐mCherry‐WPRE), hereafter referred to as SM‐MrgD, was constructed and supplied by Obio Technology Corp. Ltd. (Shanghai, China). Male SD rats (*n* = 6 per group) received a single tail vein injection of the SM‐MrgD construct at a concentration of 2 × 10^12^ viral genomes (vg)/mL. Four weeks post‐injection, rats were euthanized via cervical dislocation under isoflurane anesthesia (3.5%) for tissue collection and downstream analysis.

### 5.5 MrgD Knockdown

To evaluate the impact of MrgD downregulation on BP, male SHRs and age‐matched WKY rats (12–13 weeks old; Beijing Vital River Laboratory Animal Technology Co. Ltd.) were utilized. MrgD silencing was achieved using a recombinant adenovirus encoding MrgD‐specific shRNA (Ad‐MrgD shRNA; Genechem, Shanghai, China), while a GFP‐expressing shRNA adenovirus served as the control. The adenoviral particles (1e+7 TU/mL) were administered via tail vein injection. Tail arterial BP was recorded weekly over a 6‐week period (*n* = 8 per group). At 6 weeks, the rats were euthanized via cervical dislocation under 3.5% isoflurane anesthesia, and vascular tissues were harvested. Knockdown efficiency was confirmed by assessing MrgD expression levels.

### 5.6 MrgD Knockout Mice and Treatment

MrgD KO mice on a C57BL/6 J background were generated by Cyagen (Suzhou, China), as we previously reported [23]. Following tamoxifen administration, MrgD KO and MrgD WT mice were subjected to chronic infusion of Ang II (Sigma) or physiological saline (vehicle control) for 4 weeks. Infusions were delivered at a rate of 1.44 mg/kg/day via subcutaneously implanted osmotic pumps (ALZET model 2004; ALZET, CA, USA) (*n* = 8 per group). Tail arterial BP was measured weekly over the 4‐week infusion period. At the end of the treatment period, animals were euthanized by cervical dislocation under 2.5% isoflurane anesthesia, and tissues were harvested for analysis. Efficiency of MrgD KO was confirmed by assessing MrgD expression in relevant tissues.

### 5.7 Artery Function Assessment

Arterial function was evaluated using isolated mesenteric artery rings from MrgD‐overexpressing SD rats. Third‐order mesenteric arteries were freed of connective tissue and cut into 1 mm long rings, which were maintained in Hanks’ balanced salt solution (HBSS; Servicebio, Wuhan, China). Arterial rings were mounted on 20 µm threads in a four‐chamber myograph system (620 M; Danish Myo Technology [DMT], Denmark) and set to an initial resting tension of 0.1 g. Rings were intermittently washed every 15 min and equilibrated for 45 min prior to functional assessment. Following equilibration, each arterial ring was first exposed to a high‐potassium (K^+^) solution, composed of (mmol/L) 122.6 KCl, 1.09 CaCl_2_·7H_2_O, 1.21 MgSO_4_·7H_2_O, 1.117 KH_2_PO_4_, 24.9 KHCO_3_, and 11.1 glucose, to confirm viability, followed by washing. Subsequently, vasoconstrictive responses were elicited by cumulative concentrations of PGF2α (Sigma, CAS 4510‐16‐1) or NE (MCE, HY‐13715) over a concentration range of 10^−9^ to 10^−4^ M. Endothelium‐dependent and ‐independent vasorelaxation were assessed with Ach (MCE, HY‐B0282) and SNP (MCE, HY‐B0564), respectively, using the same concentration range (10^−9^ to 10^−4^ M).

### 5.8 Cell Culture

VSMCs were isolated from the thoracic aortas of adult male SD rats as previously described [11]. Briefly, rats were euthanized under anesthesia, and the thoracic aorta was exposed and rinsed before immersion in 75% ethanol for 2 min. The adventitia was removed, and the aorta was opened longitudinally and transferred to a digestion tube containing 2 mg/mL type I collagenase (Sigma, MO, USA). The tissue was incubated with gentle shaking at 37°C for 20 min. The partially digested vessel was then minced into 1–2 mm fragments and further incubated in Hanks’ balanced salt solution containing type I collagenase and elastase (0.5–1 mg per aorta; Sigma) at 37°C for 1–2 h until a single‐cell suspension was achieved. Cells were collected by gentle trituration, seeded in basal Dulbecco's Modified Eagle Medium/F12 (DMEM/F12; Gibco, Shanghai, China) supplemented with 15% fetal bovine serum (Gibco) and 1% penicillin/streptomycin, and cultured at 37°C in a humidified atmosphere containing 5% CO_2_. Cells were used at appropriate passage numbers for subsequent experiments.

### 5.9 VSMC Treatment

When the VSMCs in culture dishes or multiwall plates reached ∼80% confluence, they were serum‐starved in serum‐free medium for 16 h prior to treatment. Primary VSMCs at passages 4–6 were used for subsequent experiments. To induce a fibrotic phenotype, the VSMCs were treated with Ang II (10^−6^ M; Sigma). For regulating MrgD, the cells were transduced in serum‐free medium with adenoviral vectors at a multiplicity of infection of 1e+7 TU/mL: Ad‐MrgD (for overexpression; Genechem, Shanghai, China), MrgD shRNA (for knockdown; Genechem, Shanghai, China), or the corresponding negative control adenovirus.

In a separate set of experiments, the L‐type calcium channel agonist Bay K 8644 (BayK; Selleck) was applied to VSMC cultures.

### 5.10 Masson Staining

Mesenteric artery, thoracic aorta, and renal artery tissues were harvested, fixed, and sectioned into 5 µm thickness. Masson's trichrome staining (Servicebio, Wuhan, China) was performed to evaluate the extent of fibrosis in arterial tissue samples. Stained sections were examined under a light microscope (Zeiss, Germany), and digital images were acquired for quantitative analysis using Image‐Pro Plus software (Media Cybernetics Inc., Maryland, USA). For quantification, images were initially converted from 8‐bit grayscale to red‐green‐blue (RGB) color mode, followed by separation into individual color channels. The blue channel was selected as the reference. The fibrotic area was quantified as the ratio of fibrotic tissue area to the total tissue area within each section. Fibrosis‐related measurements were normalized to values obtained in the respective control group.

### 5.11 Western Blotting

Tissue and cell lysates were prepared by sonication and homogenization in RIPA buffer (Thermo Fisher Scientific, Shanghai, China). After centrifugation to remove debris, supernatants were collected. Protein samples were separated by sodium dodecyl sulfate‐polyacrylamide gel electrophoresis (SDS‐PAGE) and transferred onto polyvinylidene difluoride (PVDF) membranes. Membranes were incubated with the following primary antibodies: anti‐MrgD (1:1000; Abcam, Massachusetts, USA), anti‐α‐SMA (1:1000; Cell Signaling Technology, Danvers, MA, USA), anti‐SM‐22α (1:1000; Cell Signaling Technology), anti‐collagen I (1:1000; Abcam), and anti‐Cav1.2 (1:1000; Alomone Labs Ltd., Israel). GAPDH (1:1000; Abcam) was used as the internal loading control. Band intensities were quantified using Image‐Pro Plus software.

### 5.12 Quantitative Real‐Time PCR

Total RNA was extracted from tissue and cell samples using TRIzol reagent (Invitrogen, Thermo Fisher Scientific Inc.). Complementary DNA (cDNA) was synthesized from total RNA using the PrimeScript RT Master Mix (Takara Biotechnology Co. Ltd., Beijing, China) with random hexamer primers, following the given instructions. Each reverse transcription reaction was performed in a total volume of 10 µL, and the resulting cDNA was stored at −70°C until use. Quantitative real‐time PCR (qRT‐PCR) was conducted using SYBR Green I chemistry on a 384‐well plate format. GAPDH was used as the internal reference gene. Relative mRNA expression levels were calculated using the comparative cycle threshold (ΔCt) method. Primer sequences used for amplification are listed in Table S3.

### 5.13 Co‐Immunoprecipitation

Following protein extraction from treated cells, immune complexes were generated by incubating 1 µg of specific antibody with protein A/G agarose beads (P2055; Beyotime) at room temperature for 2 h. The antibodies used included anti‐IgG (Sc‐2027, Santa Cruz Biotechnology), anti‐Flag (F7425, Millipore Trading Co., Shanghai, China), anti‐CaMKIIγ (12666‐2‐AP, Proteintech Co., Wuhan, China), and anti‐Cav1.2 (#ACC‐013, Alomone Labs, Israel). After immune complex formation, protein lysates were added and incubated at 4°C overnight with continuous rotation. The beads were then washed three times with TBS, resuspended in SDS‐PAGE loading buffer, and subjected to electrophoresis. Immunoprecipitated proteins, including Flag, IgG, CaMKIIγ, and Cav1.2, were detected using the corresponding primary antibodies via immunoblotting.

### 5.14 Computational Virtual Screening

The three‐dimensional structure of human MrgD (UniProt ID: Q8TDS7) was predicted using the I‐TASSER homology modeling server (https://zhanglab.ccmb.med.umich.edu/I‐TASSER/). For molecular docking, a virtual screening workflow was established using AutoDock Vina (version 1.1.2) [71]. A total of 8653 compounds from the DrugBank database were screened against the modeled MrgD structure. Prior to docking, the receptor structure was converted from PDB to PDBQT format, and the docking grid was defined to encompass the full receptor surface. Protein‐ligand binding interactions were visualized and analyzed using PyMOL (version 1.7.4.5).

### 5.15 Transcription Factor Prediction

Transcription factors regulating the expression of Cav1.2 were predicted using the JASPAR database (http://jaspar.genereg.net/). A relative score threshold of ≥ 0.85 was applied, as previously described [72].

### 5.16 Statistical Analysis

All data are expressed as the mean ± standard error of the mean (SEM). The number of biological replicates (*n*) for each experimental group is indicated in the corresponding figure legends. Statistical analyses were conducted using GraphPad Prism version 8.0 (GraphPad Software Inc., CA, USA). We tested the compliance of the quantitative data with normal distribution via the Shapiro–Wilk test. The normality of data distribution was assessed prior to hypothesis testing. For comparisons between two groups, unpaired two‐tailed Student's *t*‐tests were employed. For comparisons among more than two groups, one‐way analysis of variance (ANOVA) followed by Bonferroni's post hoc test was used. The Mann–Whitney test was used to compare two groups, and the Kruskal–Wallis test was used to compare three or more groups that did not show a normal distribution. Categorical variables were analyzed using the *χ* ^2^ test. A two‐tailed *p* value of < 0.05 was considered statistically significant.

## Author Contributions

K.Z., D.H., and Y.M. performed the experiments and methodology. X.W. performed the formal analysis. M.G. provided resources. S.S. contributed to methodology. K.Z., L.C., and P.L. contributed to funding acquisition. K.Z., X.Z., and P.L. performed writing – review and editing. All authors have read and approved the final manuscript.

## Funding

This work was supported by the National Natural Science Foundation of China (82570508 and 82300309), the Academy Talent Special Fund of The First Affiliated Hospital of Nanjing Medical University (YNRCQN0312 and MXJL202208), the Jiangsu Funding Program for Excellent Postdoctoral Talent (2023ZB592), the Medical Innovation Application Research Project of Suzhou (SKY2023173), the Science Pre‐research Fund Project of the Second Affiliated Hospital of Soochow University (SDFEYGZ2221), and Science, Education, and Health Enhancement Project of Suzhou (MSXM2025014).

## Ethics Statement

All animal procedures were approved by the Experimental Animal Care and Use Committee of Nanjing Medical University (IACUC‐1906038). All human sample study protocols were approved by The First Affiliated Hospital of Nanjing Medical University (2019‐SR‐067).

## Conflicts of Interest

Author Shidong Song is an employee of Duofortunatherapeutic Suzhou Co. Ltd., but has no potential relevant financial or non‐financial interests to disclose. The other authors declare no conflicts of interest.

## Supporting information




**Figure S1**: Expression of MrgD after Ad‐MrgD treatment. A‐C, mRNA and protein levels of MrgD in the mesenteric artery (A), the thoracic aorta (B), and the renal artery (C), n = 5 biological replicates per group; The data were expressed as mean ± standard error of the mean (SEM). *P<0.05, **P<0.01, ***P<0.001, ****P<0.0001.
**Figure S2**: Expression of MrgD after MrgD‐shRNA treatment. A, mRNA levels of MrgD in the mesenteric artery, the thoracic aorta, and the renal artery, n = 6 biological replicates per group; B, protein levels of MrgD in the mesenteric artery, the thoracic aorta, and the renal artery, n = 5 biological replicates per group; The data were expressed as mean ± standard error of the mean (SEM). *P<0.05, **P<0.01, ***P<0.001, ****P<0.0001.
**Figure S3**: MrgD overexpression had no effects on remodeling of thoracic aorta. A‐B, Masson staining of the thoracic aorta or renal artery, and the quantitative analysis of fibrosis, n = 5 biological replicates per group; C, mRNA expression of α‐SMA, SM‐22α, and collagen I in Ad‐MrgDinduced mesenteric artery in SD rats, n = 5 biological replicates per group; D, mRNA expression of α‐SMA, SM‐22α, and collagen I in the thoracic aorta, n = 5 biological replicates per group; E, protein levels of α‐SMA, SM‐22α, and collagen I in the thoracic aorta, n = 5 biological replicates per group; The data were expressed as mean ± standard error of the mean (SEM). *P<0.05, **P<0.01, ***P<0.001, ****P<0.0001.
**Figure S4**: MrgD down‐regulation had no effects on remodeling of thoracic aorta. A‐B, Masson staining of the thoracic aorta or renal artery, and the quantitative analysis of fibrosis, n = 5 biological replicates per group; C, mRNA expression of α‐SMA, SM‐22α, and collagen I in MrgD shRNA‐induced mesenteric artery in SHR rats, n = 5 biological replicates per group; D, mRNA levels of α‐SMA, SM‐22α, and collagen I of thoracic aorta, n = 6 biological replicates per group; E, protein levels of α‐SMA, SM‐22α, and collagen I of the thoracic aorta, n = 5 biological replicates per group; The data were expressed as mean ± standard error of the mean (SEM). *P<0.05, **P<0.01, ***P<0.001, ****P<0.0001.
**Figure S5**: MrgD KO alleviated remodeling of the mesenteric artery. A‐B, protein and mRNA levels of MrgD in the mesenteric artery from different groups; C, mRNA levels of collagen I, α‐SMA, SM‐22α in the mesenteric artery from different groups, n = 5 biological replicates per group. The data were expressed as mean ± standard error of the mean (SEM). *P<0.05, **P<0.01, ***P<0.001, ****P<0.0001.
**Figure S6**: VSMCs‐specific overexpressed MrgD had no effects on remodeling of the renal artery and thoracic aorta. A‐B, Masson staining of the renal artery, and the quantitative analysis of fibrosis, n = 5 biological replicates per group; C‐D, Masson staining of the thoracic aorta, and the quantitative analysis of fibrosis, n = 5 biological replicates per group; The data were expressed as mean ± standard error of the mean (SEM). *P<0.05, **P<0.01, ***P<0.001, ****P<0.0001.
**Figure S7**: Expression of MrgD in the VSMCs. A‐B, mRNA and protein level of MrgD in Ad‐MrgD‐treated VSMCs; C‐D, mRNA and protein level of MrgD in MrgD shRNA‐treated VSMCs. n = 5 biological replicates per group. The data were expressed as mean ± standard error of the mean (SEM). *P<0.05, **P<0.01, ***P<0.001, ****P<0.0001.
**Figure S8**: Expression of Cav1.2 under different pathological conditions. A, MrgD overexpression increased Cav1.2 mRNA levels in the mesenteric artery of SD rats, n = 5 biological replicates per group; B‐C, mRNA levels of Cav1.2 in the thoracic aorta (A) and renal aorta (B) after Ad‐MrgD treatment, n = 5 biological replicates per group; D, MrgD knockdown reduced Cav1.2 mRNA levels in the mesenteric artery of SHR rats, n = 5 biological replicates per group; D, mRNA levels of Cav1.2 in the thoracic aorta after MrgD‐shRNA treatment, n = 6 biological replicates per group. E, mRNA levels of Cav1.2 in the renal aorta after MrgD‐shRNA treatment, n = 5 biological replicates per group. The data were expressed as mean ± standard error of the mean (SEM). *P<0.05, **P<0.01, ***P<0.001, ****P<0.0001.
**Figure S9**: Expression of Cav1.2 under different pathological conditions in VSMCs. A, Ang II or Ad‐MrgD treatment increased Cav1.2 mRNA levels in the VSMCs, n = 5 biological replicates per group; B, MrgD‐shRNA treatment reduced Ang II‐induced Cav1.2 mRNA levels in the VSMCs, n = 5 biological replicates per group. The data were expressed as mean ± standard error of the mean (SEM). *P<0.05, **P<0.01, ***P<0.001, ****P<0.0001.
**Figure S10**: Effects of 22 small molecule inhibitors on Ang II‐induced phenotypic switch of VSMCs. A, effects of 22 small molecule inhibitors on the increase of collagen I induced by MrgD overexpression in VSMCs; B, effects of 22 small molecule inhibitors on the increase of TGF‐β induced by MrgD overexpression in VSMCs; C, effects of 22 small molecule inhibitors on the increase of SM‐22α induced by MrgD overexpression in VSMCs. The data were expressed as mean ± standard error of the mean (SEM). n = 5 biological replicates per group. *P<0.05, **P<0.01, ***P<0.001, ****P<0.0001.
**Figure S11**: The expression of MrgD in endothelial cells. A, immunofluorescence co‐staining of CD31 (green) and MrgD (red) in mesenteric artery; B, endothelial expression of MrgD in both Human Umbilical Vein Endothelial cells (HUVECs) and Human Coronary Artery Endothelial cells (HCAECs).
**Table S1**: List of 22 candidate inhibitors of MrgD from FDA‐approved small molecule drugs database (2513 in DRUGBANK database, June 2021).
**Table S2**: The demographic data of enrolled patients in our study.
**Table S3**: List of utilized primers for qRT‐PCR.

## Data Availability

All data needed to evaluate the conclusion of the current study are present in the paper and/or Supporting Information. Additional data are available from the corresponding author on request.
