# Supplementary material for: Mas‐Related G Protein‐Coupled Receptor Member D Sustains Hypertension
Source: MedComm (2020). 2026 Mar 28;7(4):e70706. doi: 10.1002/mco2.70706 (PMC13042430; doi:10.1002/mco2.70706)
Supplement: Supplementary file 1 — Figure S1: Expression of MrgD after Ad‐MrgD treatment. A‐C, mRNA and protein levels of MrgD in the mesenteric artery (A), the thoracic aorta (B), and the renal artery (C), n = 5 biological replicates per group; The data were expressed as mean ± standard error of the mean (SEM). *P<0.05, **P<0.01, ***P<0.001, ****P<0.0001. Figure S2: Expression of MrgD after MrgD‐shRNA treatment. A, mRNA levels of MrgD in the mesenteric artery, the thoracic aorta, and the renal artery, n = 6 biological replicates per group; B, protein levels of MrgD in the mesenteric artery, the thoracic aorta, and the renal artery, n = 5 biological replicates per group; The data were expressed as mean ± standard error of the mean (SEM). *P<0.05, **P<0.01, ***P<0.001, ****P<0.0001. Figure S3: MrgD overexpression had no effects on remodeling of thoracic aorta. A‐B, Masson staining of the thoracic aorta or renal artery, and the quantitative analysis of fibrosis, n = 5 biological replicates per group; C, mRNA expression of α‐SMA, SM‐22α, and collagen I in Ad‐MrgDinduced mesenteric artery in SD rats, n = 5 biological replicates per group; D, mRNA expression of α‐SMA, SM‐22α, and collagen I in the thoracic aorta, n = 5 biological replicates per group; E, protein levels of α‐SMA, SM‐22α, and collagen I in the thoracic aorta, n = 5 biological replicates per group; The data were expressed as mean ± standard error of the mean (SEM). *P<0.05, **P<0.01, ***P<0.001, ****P<0.0001. Figure S4: MrgD down‐regulation had no effects on remodeling of thoracic aorta. A‐B, Masson staining of the thoracic aorta or renal artery, and the quantitative analysis of fibrosis, n = 5 biological replicates per group; C, mRNA expression of α‐SMA, SM‐22α, and collagen I in MrgD shRNA‐induced mesenteric artery in SHR rats, n = 5 biological replicates per group; D, mRNA levels of α‐SMA, SM‐22α, and collagen I of thoracic aorta, n = 6 biological replicates per group; E, protein levels of α‐SMA, SM‐22α, and collagen I of the thoracic aor [file MCO2-7-e70706-s001.pdf]

Mas-related G protein-coupled receptor member D sustains hypertension

Kun Zhao[1,#], Dongxu Hua[1,#], Yukang Mao[1,#], Xiaoguang Wu[1], Min Gao[1], Shidong Song[2], Lei Chen[3,*], Xiangxiang Zheng[4,*], Peng Li[1,5*]

[1] Department of Cardiology, the First Affiliated Hospital with Nanjing Medical University, Nanjing, Jiangsu Province, China

[2] Duofortunatherapeutic Suzhou Co., Ltd, Suzhou, Jiangsu Province, China

[3] Department of Cardiothoracic Surgery, the Second Affiliated Hospital of Soochow University, Suzhou, Jiangsu Province, China.

[4] Department of Cardiovascular Surgery, the First Affiliated Hospital of Nanjing Medical University, Nanjing, Jiangsu Province, China

[5] Department of Cardiology, Fujian Provincial Hospital, Fuzhou University Affiliated Provincial Hospital, Fuzhou, Fujian Province, China

[#]These authors contributed equally to this work.

Short Title: MrgD sustains hypertension

***Address for correspondence:**

Peng Li, Ph.D.

Department of Cardiology, the First Affiliated Hospital of Nanjing Medical University, 300 Guangzhou Road, Nanjing 210029, China

Tel: +86-25-68302826, Fax: +86-25-68302826, Email: lipeng198610@163.com

Xiangxiang Zheng, M.D.

Department of Cardiovascular Surgery, the First Affiliated Hospital of Nanjing Medical University, 300 Guangzhou Road, Nanjing 210029, China. Email: zxxford123@163.com

Lei Chen, M.D.

Department of Cardiothoracic Surgery, the Second Affiliated Hospital of Soochow University, Suzhou, Jiangsu Province, China. Email: Newheart@189.cn

Supplementary Figures

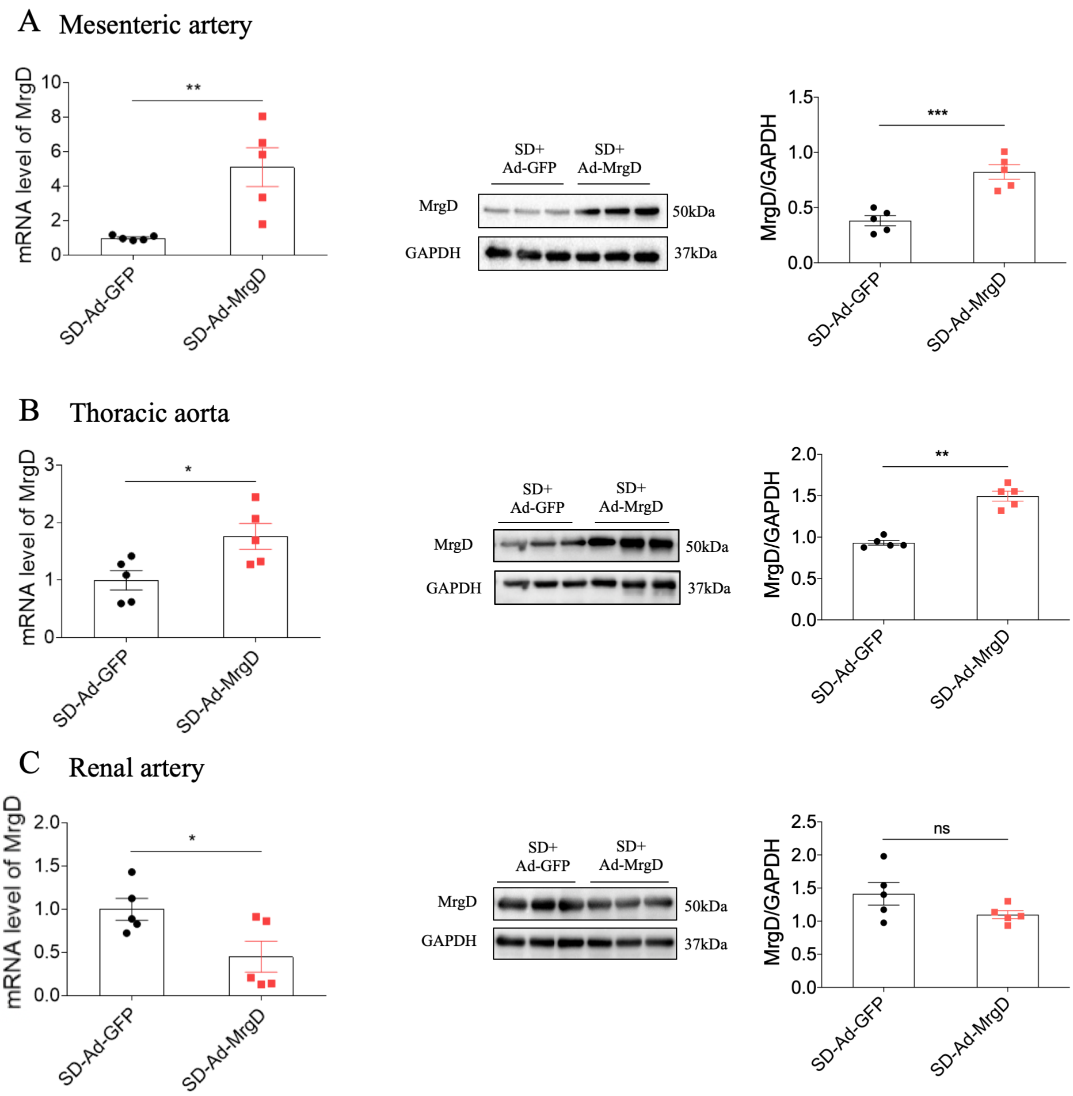

Figure S1. Expression of MrgD after Ad-MrgD treatment.

A-C, mRNA and protein levels of MrgD in the mesenteric artery (A), the thoracic aorta (B), and the renal artery (C), n=5 biological replicates per group; The data were expressed as mean ± standard error of the mean (SEM). *P<0.05, **P<0.01, ***P<0.001, ****P<0.0001.

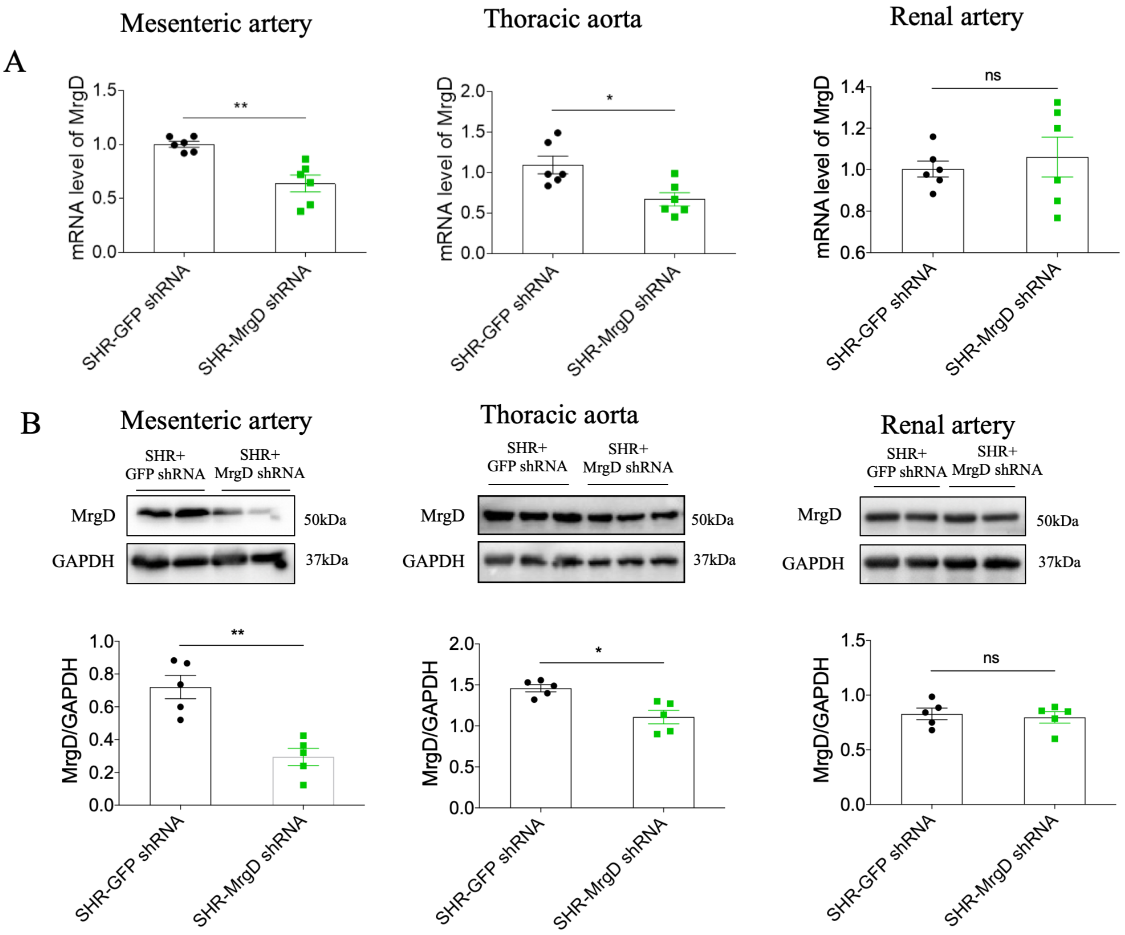

Figure S2. Expression of MrgD after MrgD-shRNA treatment.

A, mRNA levels of MrgD in the mesenteric artery, the thoracic aorta, and the renal artery, n=6 biological replicates per group; B, protein levels of MrgD in the mesenteric artery, the thoracic aorta, and the renal artery, n=5 biological replicates per group; The data were expressed as mean ± standard error of the mean (SEM). *P<0.05, **P<0.01, ***P<0.001, ****P<0.0001.

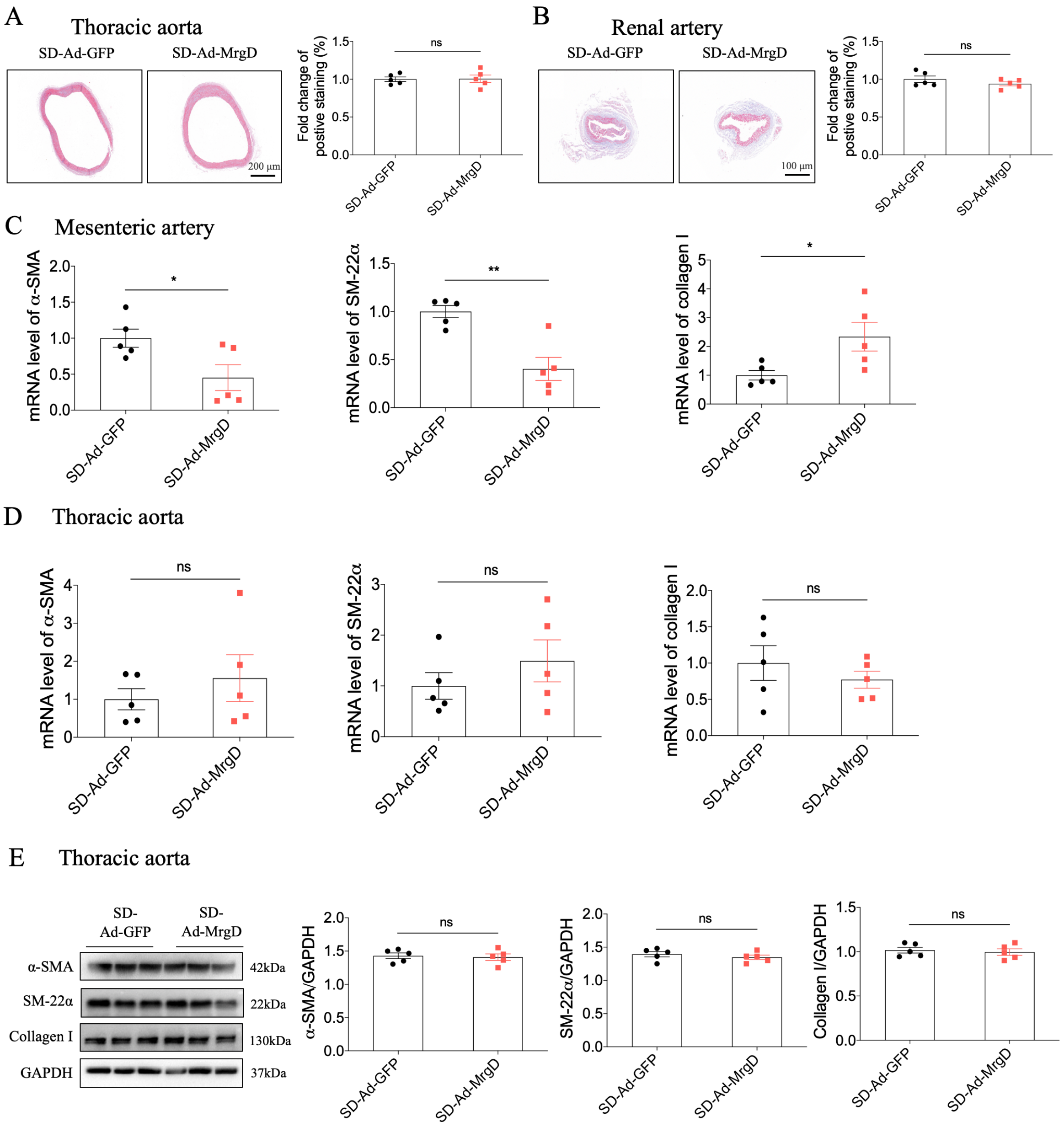

Figure S3. MrgD overexpression had no effects on remodeling of thoracic aorta.

A-B, Masson staining of the thoracic aorta or renal artery, and the quantitative analysis of fibrosis, n=5 biological replicates per group; C, mRNA expression of α-SMA, SM-22α, and collagen I in Ad-MrgD-induced mesenteric artery in SD rats, n=5 biological replicates per group; D, mRNA expression of α-SMA, SM-22α, and collagen I in the thoracic aorta, n=5 biological replicates per group; E, protein levels of α-SMA, SM-22α, and collagen I in the thoracic aorta, n=5 biological replicates per group; The data were expressed as mean ± standard error of the mean (SEM). *P<0.05, **P<0.01, ***P<0.001, ****P<0.0001.

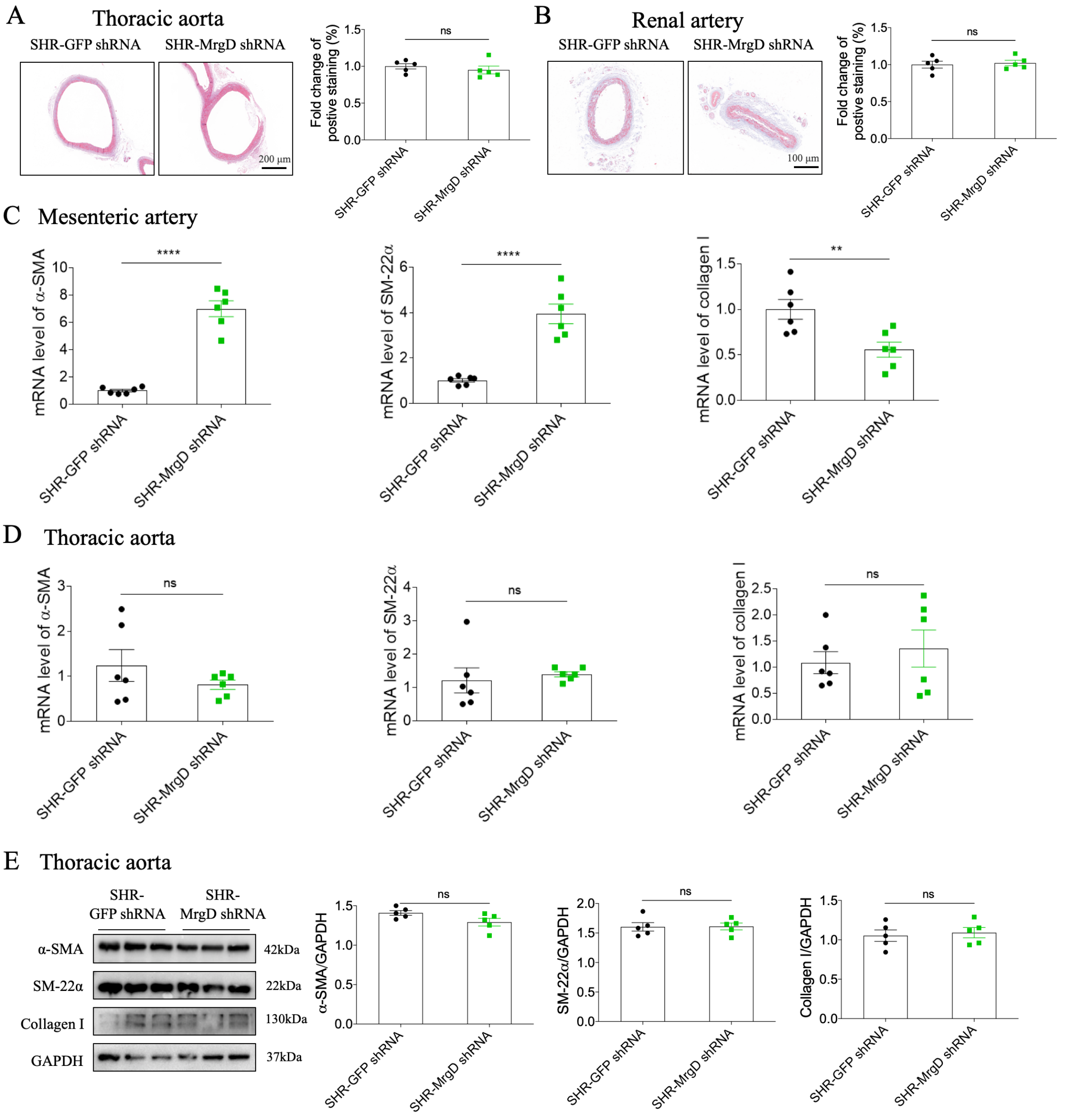

Figure S4. MrgD down-regulation had no effects on remodeling of thoracic aorta.

A-B, Masson staining of the thoracic aorta or renal artery, and the quantitative analysis of fibrosis, n=5 biological replicates per group; C, mRNA expression of α-SMA, SM-22α, and collagen I in MrgD shRNA-induced mesenteric artery in SHR rats, n=5 biological replicates per group; D, mRNA levels of α-SMA, SM-22α, and collagen I of thoracic aorta, n=6 biological replicates per group; E, protein levels of α-SMA, SM-22α, and collagen I of the thoracic aorta, n=5 biological replicates per group; The data were expressed as mean ± standard error of the mean (SEM). *P<0.05, **P<0.01, ***P<0.001, ****P<0.0001.

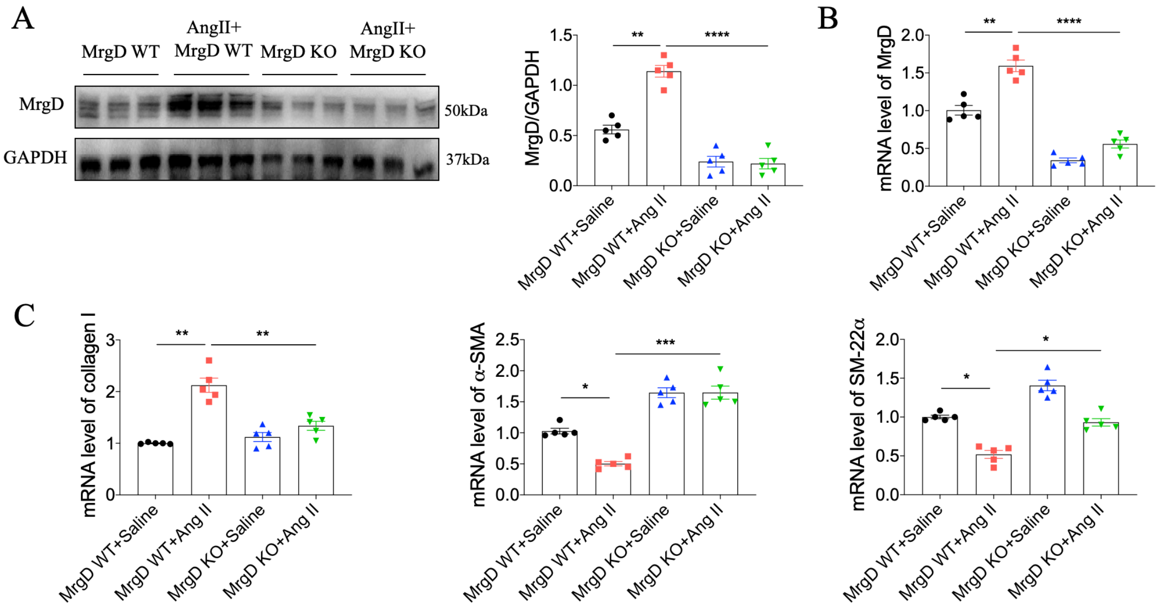

Figure S5. MrgD KO alleviated remodeling of the mesenteric artery.

A-B, protein and mRNA levels of MrgD in the mesenteric artery from different groups; C, mRNA levels of collagen I, α-SMA, SM-22α in the mesenteric artery from different groups, n=5 biological replicates per group. The data were expressed as mean ± standard error of the mean (SEM). *P<0.05, **P<0.01, ***P<0.001, ****P<0.0001.

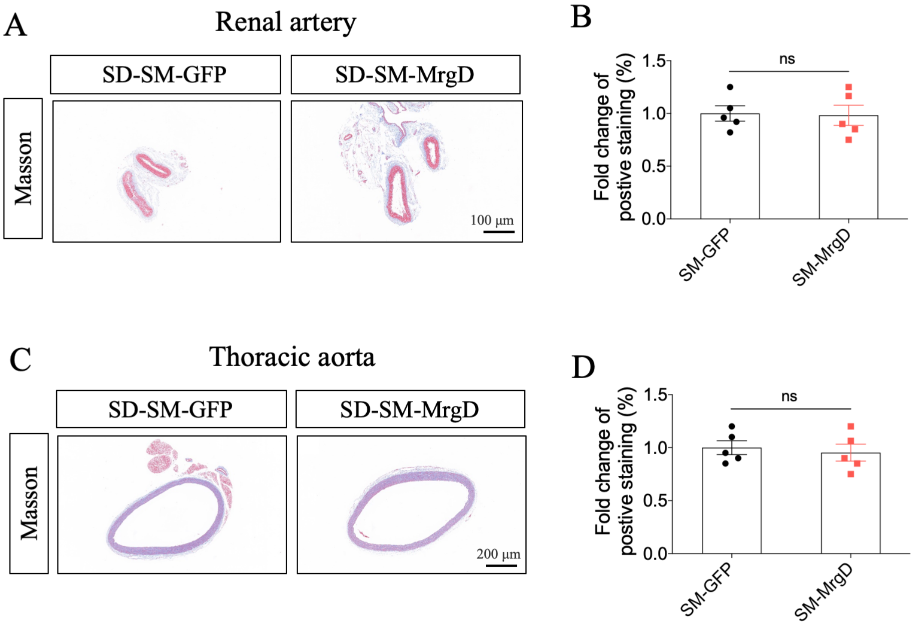

Figure S6. VSMCs-specific overexpressed MrgD had no effects on remodeling of the renal artery

and thoracic aorta.

A-B, Masson staining of the renal artery, and the quantitative analysis of fibrosis, n=5 biological replicates per group; C-D, Masson staining of the thoracic aorta, and the quantitative analysis of fibrosis, n=5 biological replicates per group; The data were expressed as mean ± standard error of the mean (SEM). *P<0.05, **P<0.01, ***P<0.001, ****P<0.0001.

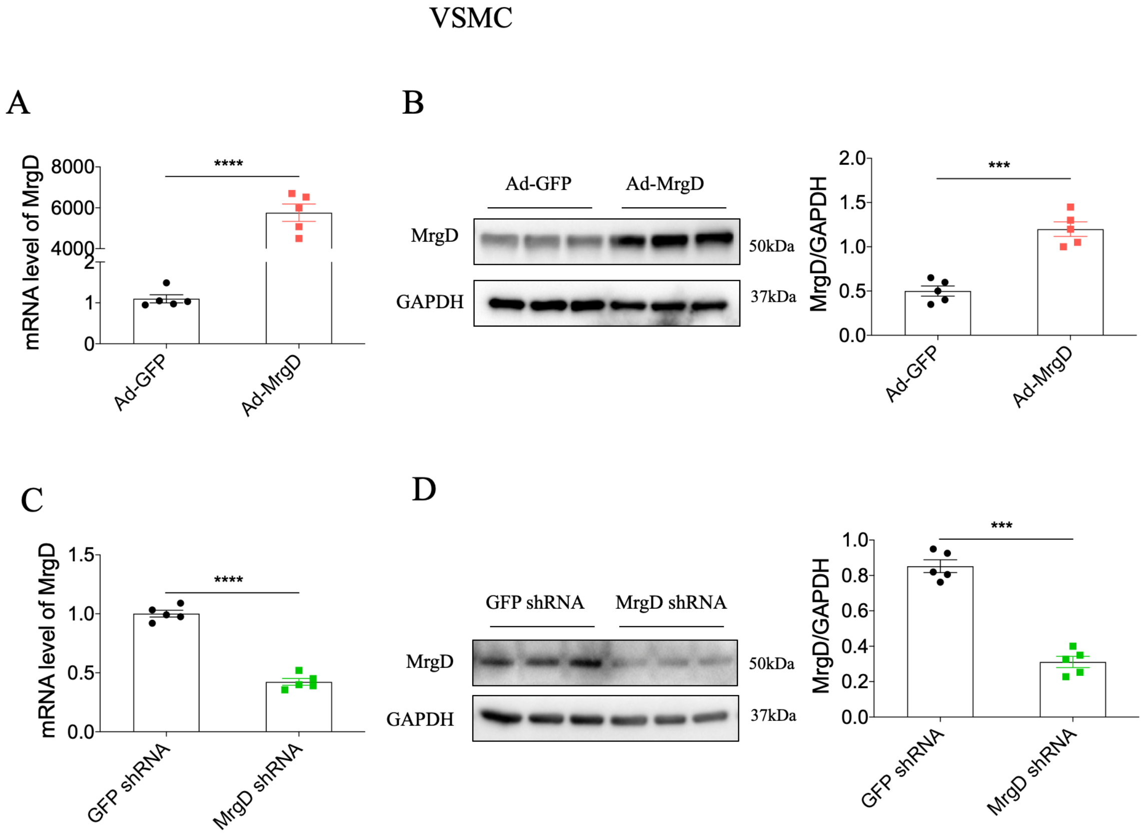

Figure S7. Expression of MrgD in the VSMCs.

A-B, mRNA and protein level of MrgD in Ad-MrgD-treated VSMCs; C-D, mRNA and protein level of MrgD in MrgD shRNA-treated VSMCs. n=5 biological replicates per group. The data were expressed as mean ± standard error of the mean (SEM). *P<0.05, **P<0.01, ***P<0.001, ****P<0.0001.

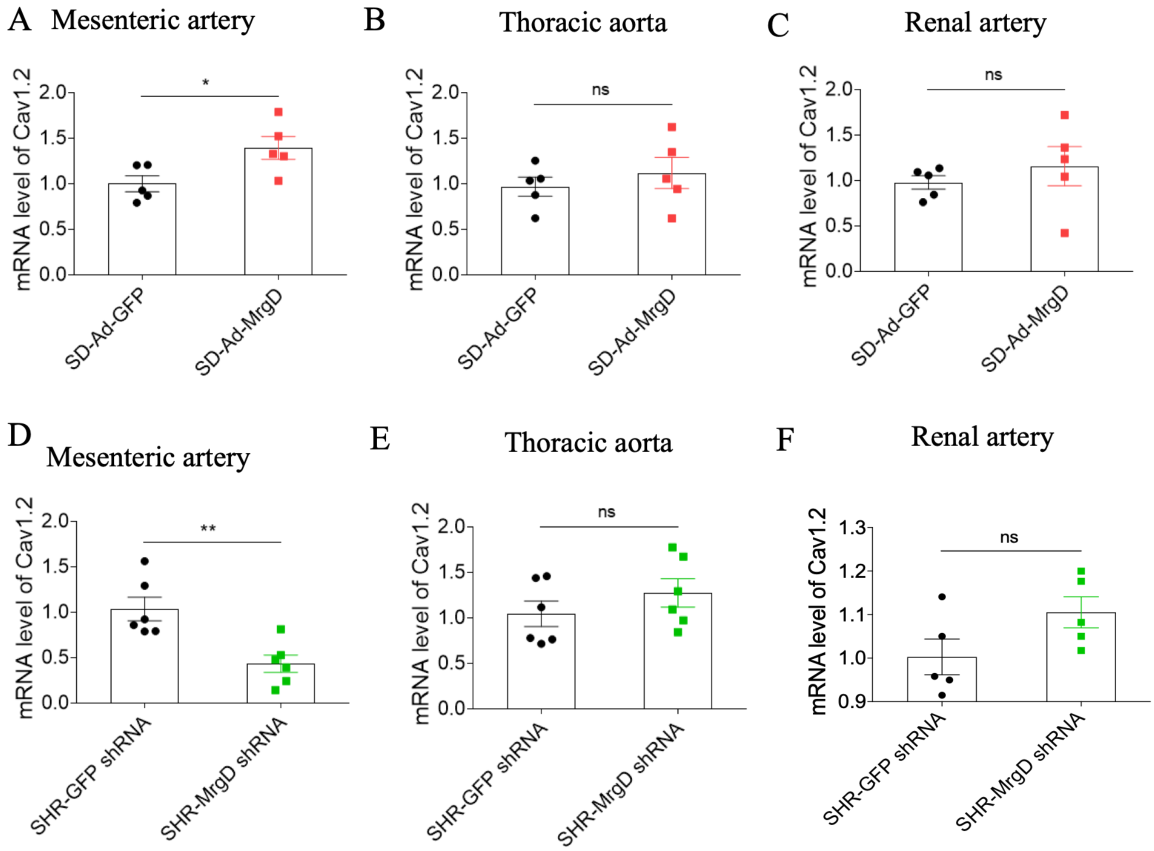

Figure S8. Expression of Cav1.2 under different pathological conditions.

A, MrgD overexpression increased Ca$_v$1.2 mRNA levels in the mesenteric artery of SD rats, n=5 biological replicates per group; B-C, mRNA levels of Cav1.2 in the thoracic aorta (A) and renal aorta (B) after Ad-MrgD treatment, n=5 biological replicates per group; D, MrgD knockdown reduced Ca$_v$1.2 mRNA levels in the mesenteric artery of SHR rats, n=5 biological replicates per group; D, mRNA levels of Cav1.2 in the thoracic aorta after MrgD-shRNA treatment, n=6 biological replicates per group. E, mRNA levels of Cav1.2 in the renal aorta after MrgD-shRNA treatment, n=5 biological replicates per group. The data were expressed as mean ± standard error of the mean (SEM). *P<0.05, **P<0.01, ***P<0.001, ****P<0.0001.

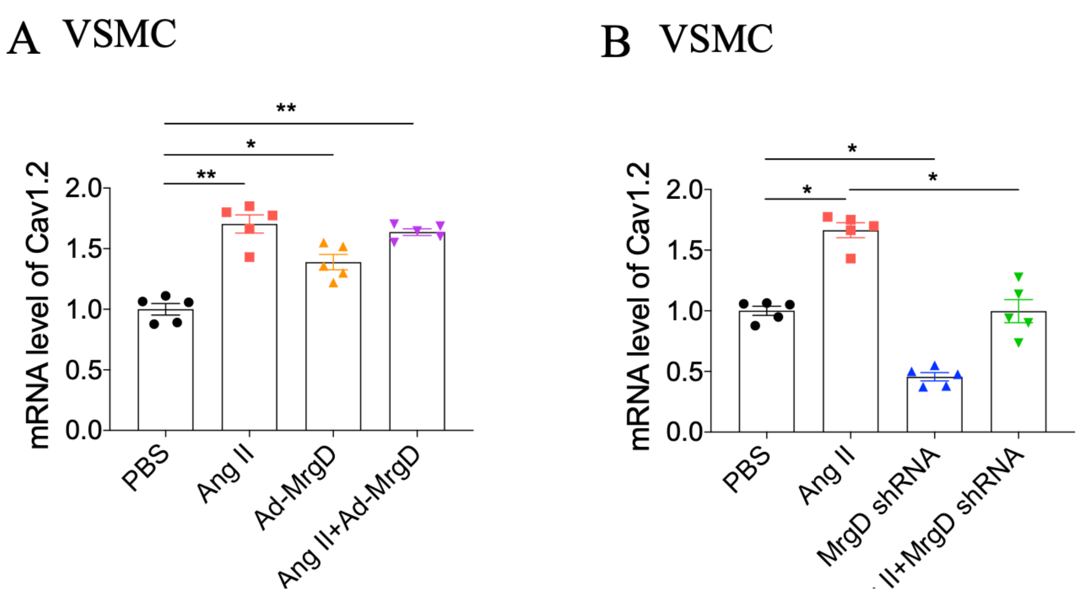

Figure S9. Expression of Cav1.2 under different pathological conditions in VSMCs.

A, Ang II or Ad-MrgD treatment increased Ca_v1.2 mRNA levels in the VSMCs, n=5 biological replicates per group; B, MrgD-shRNA treatment reduced Ang II-induced Ca_v1.2 mRNA levels in the VSMCs, n=5 biological replicates per group. The data were expressed as mean ± standard error of the mean (SEM). *P<0.05, **P<0.01, ***P<0.001, ****P<0.0001.

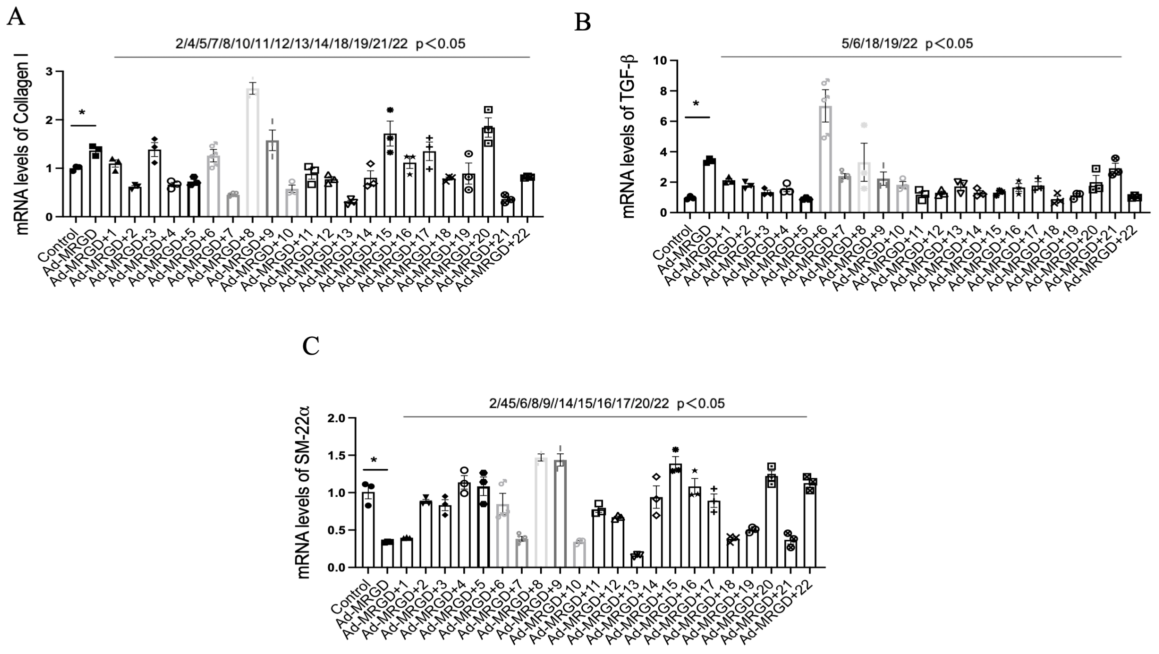

Figure S10. Effects of 22 small molecule inhibitors on Ang II-induced phenotypic switch of VSMCs.

A, effects of 22 small molecule inhibitors on the increase of collagen I induced by MrgD overexpression in VSMCs; B, effects of 22 small molecule inhibitors on the increase of TGF-β induced by MrgD overexpression in VSMCs; C, effects of 22 small molecule inhibitors on the increase of SM22α induced by MrgD overexpression in VSMCs. The data were expressed as mean ± standard error of the mean (SEM). n=5 biological replicates per group. *P<0.05, **P<0.01, ***P<0.001, ****P<0.0001.

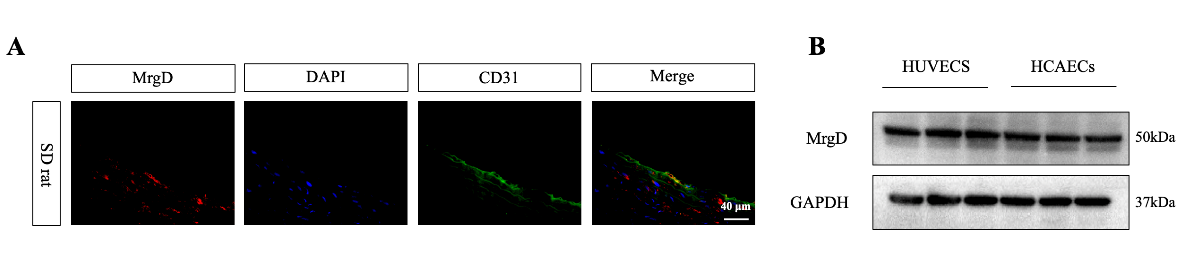

Figure S11. The expression of MrgD in endothelial cells.

A, immunofluorescence co-staining of CD31 (green) and MrgD (red) in mesenteric artery; B, endothelial expression of MrgD in both Human Umbilical Vein Endothelial cells (HUVECs) and Human Coronary Artery Endothelial cells (HCAECs).

Table S1: List of 22 candidate inhibitors of MrgD from FDA-approved small molecule drugs database (2513 in DRUGBANK database, June 2021)

Number	Drug name
1	Vorapaxar
2	Zafirukast
3	Irinotecan
4	Hydrocortisone
5	Ponatinib
6	Ciclesonide
7	Astemizole
8	Bisoctrizole
9	Delamanid
10	Pimozide
11	Telmisartan
12	NVP-LDE225
13	Nilotinib
14	Dutasteride
15	Nandrolone phenylpropionate
16	lumacaftor
17	lurasidone
18	Gliquidone
19	Enasidenib
20	Novobiocin Sodium
21	Ebastine
22	Risperidone

Table S2. The demographic data of enrolled patients in our study.

Characteristic	Norm (n=5)	HTN (n=5)
Age (years)	59.20±4.19	58.00±5.54
Sex (male/female)	3/2	4/1
SBP(mm Hg)	116.4±3.04	159.2±4.53
DBP(mm Hg)	61.00±1.95	94.80±8.24
MBP(mm Hg)	79.47±1.86	116.3±6.95
Height (cm)	167.2±3.84	169.2±4.73
Weight (kg)	68.00±3.52	71.00±5.96
Complication		
Diabetes (%)	1 (20.0%)	3 (60.0%)
Coronary heart disease (%)	0 (0.0%)	1 (20.0%)
Diabetic Nephropathy (%)	0 (0.0%)	0 (0.0%)

Norm, Normal patients; HTN, Hypertension patients; SBP, systolic blood pressure; DBP, diastolic blood pressure; MAP, mean blood pressure.

Table S3. List of utilized primers for qRT-PCR.

Gene	Species	Forward primer	Reverse primer
Cav1.2	Rat	CCTGCTGGTGGTTAGCGTG	TCTGCCTCCGTCTGTTTAGAA
SM-22α	Rat	CAACAAGGGTCCATCCTACGG	ATCTGGGCGGCCTACATCA
α-SMA	Rat	GCATCCACGAAACCACCTA	CACGAGTAACAAATCAAAGC
TGF-β	Rat	TCTGCATTGCACTTATGCTGA	AAAGGGCGATCTAGTGATGGA
Collagen I	Rat	GCTCCTCTTAGGGGCCACT	CCACGTCTCACCATTGGGG
MrgD	Rat	CACTGGCCCTCCTGATGAA	GGATGCCAGAATTGAACACAGA
GAPDH	Rat	GGCACAGTCAAGGCTGAGAATG	ATGGTGGTGAAGACGCCAGTA
